# Fabrication and Wear Performance of Al Matrix Composites Reinforced with Metallic and Oxidized WMoNb Medium-Entropy Alloy Powders

**DOI:** 10.3390/ma19173692

**Published:** 2026-08-30

**Authors:** Muhammet Gökhan Albayrak

**Affiliations:** Facility of Engineering, Department of Metallurgy and Materials Engineering, Firat University, 23240 Elazığ, Türkiye; mgalbayrak@firat.edu.tr

**Keywords:** medium-entropy alloy, WMoNb, oxidation, core–shell, wear behavior, refractory alloy

## Abstract

**Highlights:**

- Equiatomic WMoNb medium-entropy alloy (RMEA) powder was synthesized by 150 h high-energy ball milling into a single-phase BCC solid solution (~22 nm crystallite size).
- Selective oxidation (650 °C/4 h) converted Mo and Nb into Mo_4_O_11_/NbO_0.76_ while W stayed metallic, forming a novel core–shell oxide reinforcement (RMEO).
- RMEO-reinforced Al composites reached the highest hardness (132 HB) and lowest coefficient of friction (0.192) and wear loss (52 mg) of all compositions.
- At 10 wt.%, RMEO cut friction and wear loss by ~60% vs. pure Al, outperforming unoxidized RMEA (53% and ~48% reduction) at every ratio.
- Wear mode shifted from severe adhesive wear in pure Al to a protective tribo-oxide-mediated regime in RMEO composites, confirming oxidation as a deliberate design strategy.

**Abstract:**

Aluminum matrix composites reinforced with refractory medium-entropy alloy particles are of growing interest for wear-resistant applications, yet the contribution of an oxidized core–shell reinforcement remains largely unexplored. This study investigates the fabrication and tribological performance of Al composites reinforced with WMoNb refractory medium-entropy alloy (RMEA) powder and its oxidized derivative (RMEO). Equiatomic WMoNb powder was synthesized by 150 h high-energy ball milling, forming a single-phase BCC solid solution, then oxidized at 650 °C/4 h, selectively converting Mo and Nb into Mo_4_O_11_ and NbO_0.76_ while W remained metallic, yielding a core–shell RMEO structure. Composites containing 2.5–10 wt.% RMEA or RMEO were fabricated by cold pressing/sintering and evaluated by dry sliding wear testing (pin-on-disc, Al_2_O_3_ counterpart). Both reinforcements reduced friction and wear loss relative to pure Al in a dose-dependent manner; RMEO outperformed RMEA at every ratio, cutting friction and wear loss by ~60% at 10 wt.% and reaching the highest hardness (132 HB) of all compositions. Worn-surface and profilometric analyses revealed a wear-mechanism shift from severe adhesive/abrasive wear in pure Al to a tribo-oxide-mediated regime in RMEO composites. These findings indicate that selective oxidation of refractory medium-entropy alloy powders is an effective strategy for enhancing the wear resistance of Al matrix composites.

## 1. Introduction

One of the most striking manifestations of the paradigm shift that has taken place in the field of engineering materials over the past two decades is the concept of multi-principal element alloy design. The conventional alloy development approach, which relied on compositional strategies built around a single dominant element, was fundamentally challenged by the high-entropy alloy (HEA) concept independently proposed by Yeh et al. [1] and Cantor et al. in 2004 [2]. According to the HEA concept, the high mixing entropy (ΔS_~mix~_ ≥ 1.5 R) achieved by combining at least five principal elements in near-equiatomic ratios can promote the stabilization of single-phase solid solutions under both equilibrium and non-equilibrium conditions, rather than leading to the formation of multi-phase microstructures or intermetallic compounds as would conventionally be expected [3,4]. This alloy family has been extensively documented in the literature as a class of material systems capable of simultaneously delivering multiple superior properties, including high strength, exceptional hardness, structural stability over a wide temperature range, fatigue and corrosion resistance, and outstanding wear performance [4].

As a natural extension of the expanding HEA literature, medium-entropy alloys (MEAs) [4]—in which three to four principal elements are combined in near-equiatomic ratios, yielding mixing entropy values in the range of 0.69R to 1.61R—have attracted increasing attention. While MEA systems offer compositional design flexibility with a more limited elemental diversity compared to HEAs, they frequently exhibit similar or complementary mechanical properties, thereby establishing themselves as an alternative multi-principal element alloy platform for both structural and functional applications [5]. In this context, HEA and MEA systems incorporating refractory elements have risen to considerable prominence in applications involving demanding service conditions—such as aerospace, nuclear, energy, and heavy industry—owing to their superior thermal stability, high hardness, and favorable mechanical properties conferred by high-melting-point elements including W, Mo, Nb, Ta, and Hf [3,6,7,8,9]. Refractory alloy systems containing W–Mo–Nb are capable of retaining their body-centered cubic (BCC) crystal structure over wide temperature ranges, constituting a strong alternative to conventional nickel-based superalloys in terms of high-temperature creep resistance, mechanical robustness, and dimensional stability. In particular, four- to five-component refractory HEA systems such as NbMoTaW and WMoTaNbV have been fabricated via advanced powder metallurgy techniques including spark plasma sintering (SPS) and laser powder bed fusion (PBF-LB/M), and these alloys have been demonstrated to possess critical properties such as high microhardness, thermal stability, and resistance to thermal roughening [10]. Nevertheless, studies addressing the ternary W–Mo–Nb system within the medium-entropy alloy framework—with a specific focus on powder fabrication and characterization—remain considerably limited in the open literature. Aluminum and its alloys have found widespread application across the automotive, aerospace, construction, and electrical-electronics industries owing to their low density (~2.7 g/cm^3^), high specific strength, and favorable machinability [11,12,13]. However, the inherent limitations of aluminum, namely its low hardness and insufficient wear resistance, impose a significant performance constraint, particularly in components operating under high loads and frictional conditions. To overcome this shortcoming, conventional reinforcement phases such as ceramics (SiC, Al_2_O_3_), graphene, and carbon nanotubes have long been investigated [14,15]; however, these reinforcements introduce serious limitations, including interfacial incompatibility with the matrix, a tendency toward clustering, and the a of forming brittle interfaces. Against this background, the use of MEA particles as reinforcing phases in metal matrix composites has emerged as a novel and increasingly significant approach in recent years [16]. Studies on HEA particle-reinforced Al matrix composites reported in the literature have yielded promising results. It has been reported that Al composites reinforced with HEA particles and fabricated via cold spraying and friction stir processing (FSP) exhibit markedly improved corrosion and wear resistance compared to the pure Al matrix [17]. Similarly, in HEA-reinforced Al matrix composites produced by pulsed electric current sintering (PECS), a 10 wt.% HEA addition has been reported to increase microhardness by approximately 178% while significantly reducing the coefficient of friction [18].

In the context of MEA-reinforced Al composites, research conducted on CrCoNi-reinforced Al matrix composites has demonstrated that MEA particles are capable of forming strong interfacial bonds with the matrix, thereby yielding significant improvements in both strength and wear resistance [19,20,21]. These developments suggest that MEA particles may offer a more advantageous interfacial chemistry and compatibility compared to ceramic reinforcements, particularly for wear-critical Al matrix composite applications. Nevertheless, no systematic study addressing the use of W–Mo–Nb-based refractory medium-entropy alloy powders as a reinforcing phase in Al matrix composites has been identified in the open literature; existing works have predominantly focused on four- to five-component refractory HEA compositions such as WMoTaNb, or on the tribological behavior of MEA-containing coatings.

In addition to metallic HEA and MEA particles, recent studies highlight that controlled oxidation of multiple parent element reinforcements can be used to create heterogeneous core–shell architectures and thus shape the interface chemistry and stress distribution in Al matrix composites. In oxidized HEA/Al systems, thin, compositionally complex oxide layers formed at the particle surface have been shown to promote improved wettability, refined interfacial microstructures, and enhanced strength–ductility synergy by modulating local stress gradients and inhibiting crack propagation [22]. Owing to this combination of high specific strength, improved wear and corrosion resistance, and tunable interfacial toughness, oxidized HEA- or MEA-reinforced Al composites are increasingly being considered for lightweight structural components in aerospace and automotive sectors, wear-critical parts in transportation and energy systems, and corrosion-resistant architectures in marine and chemical-processing environments [23]. Building on these insights, the W–Mo–Nb medium-entropy alloy system offers a promising platform for the design of refractory MEA powders with a deliberately oxidized shell, where a WMoNb-oxide layer could act as a chemically compatible, mechanically robust interphase between the metallic RMEA core and the Al matrix. Such an approach is expected to combine the high hardness and thermal stability of the refractory MEA core with the potential benefits of a tailored oxide interlayer in terms of wear resistance and interfacial integrity under demanding tribological loading.

Despite the growing body of work on HEA- and MEA-reinforced Al composites, systematic investigations that explicitly address the role of surface oxidation in refractory multi-principal element reinforcements remain limited, and existing studies predominantly focus on transition-metal HEA particulates without a well-defined oxide shell. In particular, no comprehensive study has been reported on W–Mo–Nb-based refractory MEA powders in which the formation, composition, and thickness of a WMoNb-oxide layer are controlled and correlated with the tribological response of Al matrix composites, including wear rate, coefficient of friction, and worn surface morphology. This gap suggests that introducing and characterizing WMoNb-oxide as a functional interfacial phase in Al–WMoNb composites could provide new insights into how refractory MEA core–shell architectures govern microstructure–tribology relationships, and may open a pathway toward next-generation wear-resistant Al-based materials with optimized interfacial chemistry and multi-scale heterogeneity.

Motivated by this research gap, the present study aims to fabricate W–Mo–Nb medium-entropy alloy powders under controlled conditions, both in their metallic (RMEA) and selectively oxidized (RMEO) forms, and to investigate the effect of the resulting powders on the tribological behavior of Al matrix composites in which they serve as reinforcing phases. To this end, the effects of powder production and oxidation parameters on the morphology and phase composition of RMEA and RMEO powders will be examined; subsequently, the wear rate, coefficient of friction, and worn surface characteristics of Al–RMEA and Al–RMEO composites will be systematically evaluated through pin-on-disc testing. The present work is expected to provide an original contribution by elucidating the relationships between RMEA/RMEO powder design and tribological performance.

## 2. Materials and Methods

### 2.1. Raw Material Selection and Powder Preparation

In this study, high-purity (≥99.99%) commercially available W, Mo, and Nb elemental powders were used. Similarly, Al powders (Al ≥ 99%) were also purchased commercially. The WMoNb RMEA composition was designed by combining the three elements in equiatomic ratios (33.3 at.% W—33.3 at.% Mo—33.3 at.% Nb). The elemental powders were weighed (Shimadzu AUW220D, Okayama, Japan) precisely in the designated proportions and prepared for the blending process.

### 2.2. Mechanical Alloying Process

High-energy ball milling (HEBM) was employed for the fabrication of WMoNb RMEA powders. The milling process was carried out in stainless steel vials and 6 mm diameter stainless steel balls under nitrogen (N ≥ 99.99%) atmosphere at a fixed ball-to-powder weight ratio (BPR) of 15:1 for milling for 150 h. To prevent excessive temperature rise and cold welding of the powders during milling, a 15 min cooling pause was applied after every 15 min of milling.

### 2.3. Oxidation Process

An open-air muffle furnace was used for the oxidation process of RMEA powders produced with MA. The oxidation temperature was set at 650 °C. The powders were mechanically mixed every 30 min for 4 h to obtain highly homogenous oxides. The heating rate was selected as 5 °C/min and the powders were cooled at room temperature after 4 h heating.

### 2.4. Powder Characterization

The morphological, chemical, and structural properties of the produced WMoNb RMEA powders were examined using the following techniques:

- Phase Analysis: Phase composition and crystal structure were determined by X-ray diffraction (XRD, Rigaku with Cu Kα radiation, Tokyo, Japan) using Cu-Kα radiation over a 2θ range of 10–90°.
- Morphology and Composition: Powder morphology, particle size, and elemental distribution were analyzed by scanning electron microscopy (SEM, Hitachi SU3500 (Tokyo, Japan) with an acceleration voltage of 15 kV) coupled with energy-dispersive X-ray spectroscopy (EDS, Oxford AZtech, High Wycombe, UK) and EDS mapping.

### 2.5. Composite Fabrication

Commercial-purity aluminum powder (Al, ≥ 99.9%, average particle size ~45 µm) was used as the matrix material, and WMoNb RMEA powders were incorporated into the Al matrix at weight fractions of 0, 2.5, 5, 7.5, and 10 wt.%. The powder mixtures were blended in a low-energy mixer for 2 h at 100 rpm to ensure homogeneous distribution. The blended mixtures were subsequently compacted into cylindrical specimens (10 mm diameter) by uniaxial cold pressing at 600 MPa and sintered at 550 °C for 2 h under an argon atmosphere.

### 2.6. Wear Testing

The tribological properties of the fabricated Al–WMoNb composite specimens were evaluated using a pin-on-disc tribometer under dry sliding conditions. The test parameters were as follows:

Counterpart material: Al_2_O_3_ ball (diameter: 6 mm, hardness: ~1800 HV);

Applied load: 10 N;

Sliding speed: 0.1 m/s;

Total sliding distance: 1000 m;

Ambient temperature: 25 ± 2 °C;

Relative humidity: 45 ± 5%;

Number of replicate tests: 1.

Prior to each test, specimen surfaces were ground with 1200-grit abrasive paper and subsequently cleaned ultrasonically in acetone. The wear loss (mg) was calculated using the mass loss method (precision: g^−4^). Worn surfaces were examined by SEM to identify the dominant wear mechanisms (abrasive, adhesive, oxidative, etc.).

## 3. Results

### 3.1. Characterization of RMEA

#### XRD Results and Solid Solution Formation

**Figure 1:**
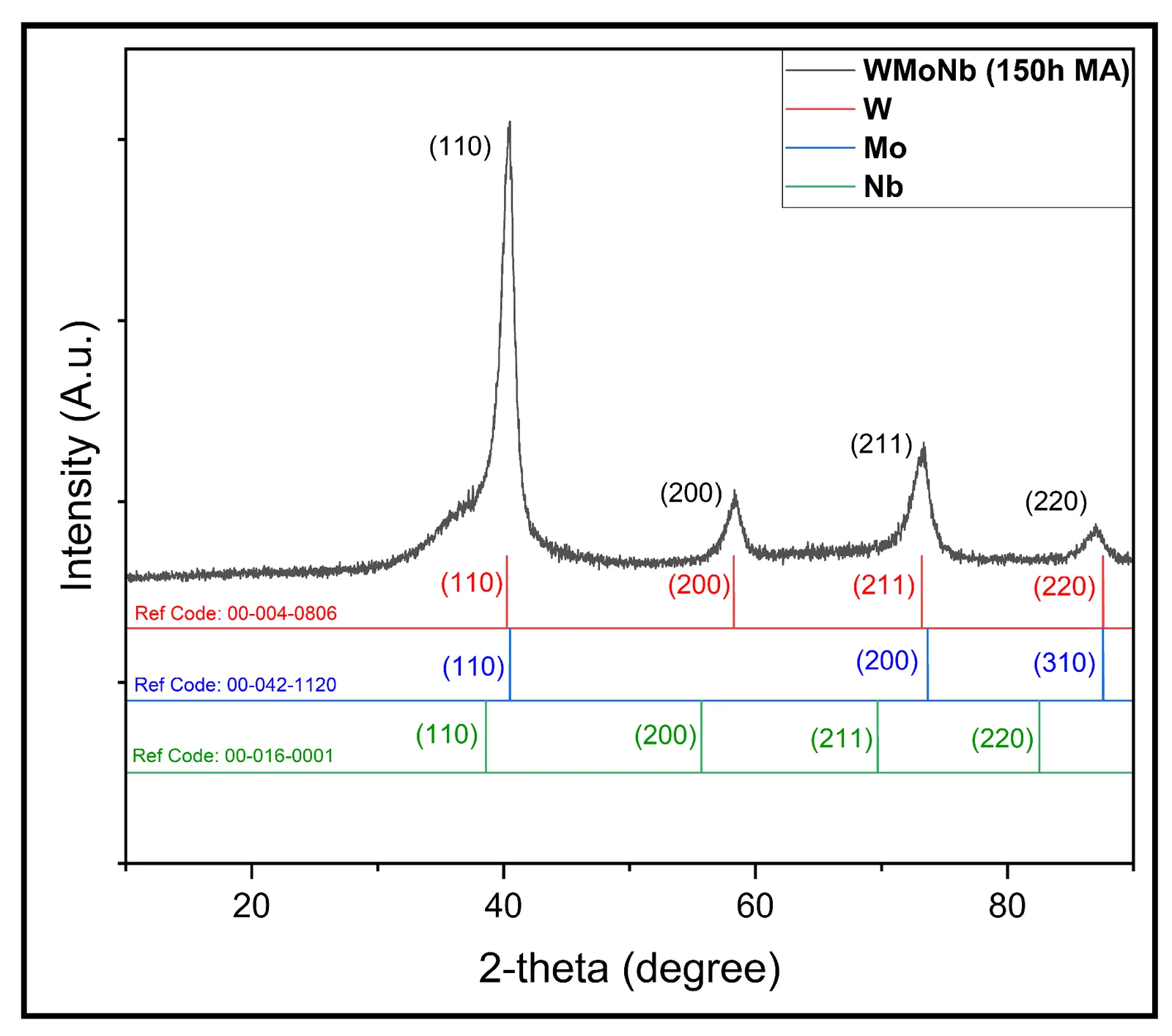
XRD analysis of WMoNb powder after 150 h of MA.

The XRD pattern of the WMoNb RMEA powder after 150 h of mechanical alloying (MA) is presented in Figure 1, along with the reference diffraction patterns of the constituent elemental powders W (ICDD: 00-004-0806), Mo (ICDD: 00-042-1120), and Nb (ICDD: 00-016-0001). The diffractogram revealed a single-phase body-centered cubic (BCC) solid solution, as evidenced by the presence of four characteristic diffraction peaks indexed to the (110), (200), (211), and (220) crystallographic planes. Importantly, no diffraction peaks corresponding to intermetallic compounds, oxides, or secondary phases were detected.

The thermodynamic parameters of the equiatomic WMoNb MEA were calculated to assess its phase formation tendency.

$$\mathrm{Mixing}\;\mathrm{Entropy}:\;\Delta S_{\mathrm{mix}}=-R\;\sum_{i=1}^{n}c_{i}\ln c_{i}$$

$$\mathrm{Mixing}\;\mathrm{Enthalpy}:\;\Delta H_{\mathrm{mix}}=\sum_{i\neq j}4∆H_{ij}^{mix}.c_{i}c_{j}$$

The mixing entropy [24] was calculated as ΔS_mix_ = 9.13 J/(mol·K) (1.099 R), which falls within the medium-entropy alloy range (0.69 R–1.61 R), confirming the MEA classification of the WMoNb system. The mixing enthalpy [24] was found to be ΔH_mix_ = −4.44 kJ/mol, indicating mild negative mixing interactions that favor solid solution formation without promoting intermetallic compound precipitation.

A pronounced broadening of all diffraction peaks was observed in the WMoNb MEA pattern relative to the sharp reference peaks of the individual elemental powders.

To quantitatively separate the contributions of crystallite size to the total peak broadening, the Scherrer equation [25] was applied using the following linear equation:

D=Kλ/(βcosθ),

where:

β is the full width at half maximum (FWHM) of the diffraction peak (in radians);

θ is the Bragg diffraction angle;

K is the Scherrer shape factor (typically 0.9 for spherical particles);

λ is the X-ray wavelength (Cu-Kα: 0.15406 nm);

D is the mean crystallite size.

After 150 h of grinding, the estimated average crystallite size of WMoNb MEA was calculated to be approximately 22.1 nm (Table 1).

**Figure 2:**
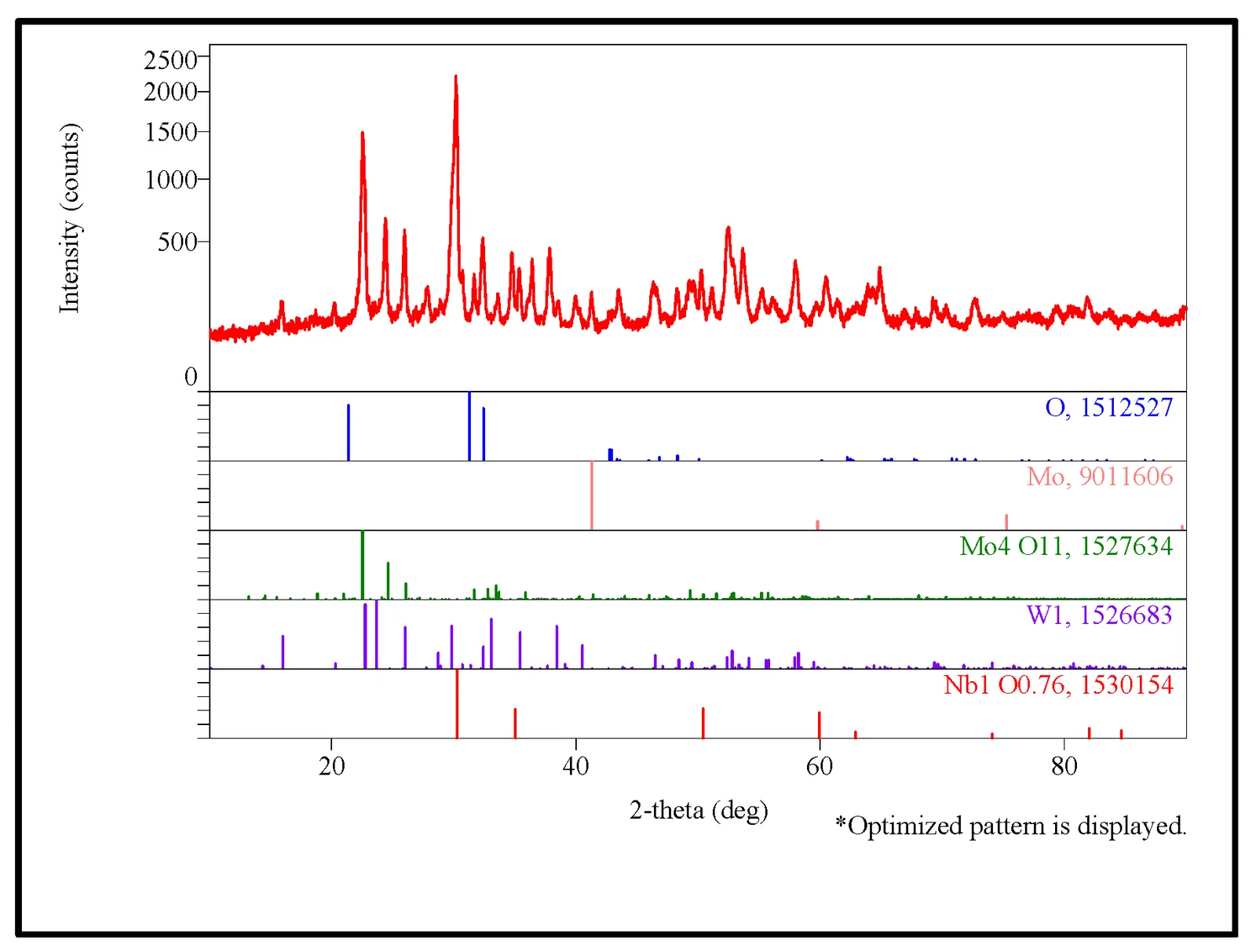
XRD analysis of WMoNb powder oxidized at 650 °C for 4 h after 150 h of MA.

The XRD pattern of WMoNb refractory powder (in Figure 2), oxidized at 650 °C for 4 h after 150 h of MA, shows a heterogeneous structure where the powder emerges from a completely amorphous/nanocrystalline BCC solid solution, with W (DB Card Number: 1526683) remaining largely metallic, while Mo and Nb are selectively converted into substoichiometric oxides (Mo_4_O_11_—DB Card Number: 1527634 and NbO_0.76_—DB Card Number: 1530154).

The diffractogram shows a broad, dome-shaped background (broad hump) in the range of 2θ ≈ 20–35°, superimposed by two narrow and intense peaks (≈23° and ≈31°).

Reading these two XRD analyses sequentially, the process can be summarized as shown in Table 2.

**Figure 3:**
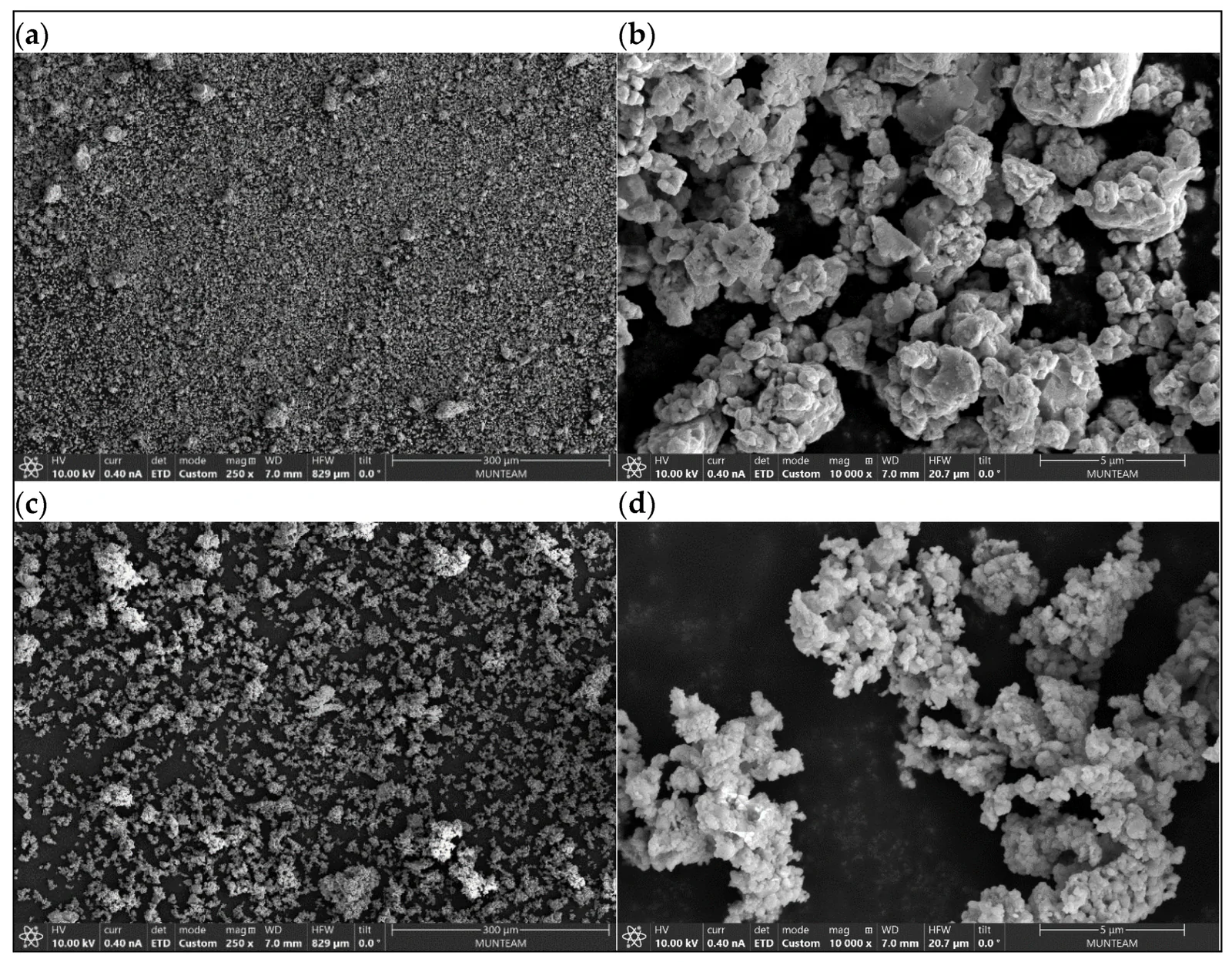
SEM images of WMoNb powder at (**a**) 250× magnification and (**b**) 10,000× magnification, and oxide powder at (**c**) 250× magnification and (**d**) 10,000× magnification.

The SEM images of the 150 h mechanically alloyed WMoNb and 650 °C/4 h oxide powder at two different magnifications are shown in Figure 3.

A low-magnification SEM image Figure 3a displays the general morphology of typical mechanically alloyed WMoNb powder. The observed area forms a nearly continuous powder bed densely coated with fine particles distributed across the entire surface. While most of the particles appear relatively small, irregular, and semi-circular in shape, a limited number of larger agglomerates are also present.

Based on the scale bar in the figure, the majority of the fine particles appeared to be in the sub-micron to a few micrometers range, whereas the larger agglomerates reached several tens of micrometers in size.

On the other hand, high-magnification SEM imaging Figure 3b provides more detailed information about the surface and microstructural properties of WMoNb powder particles. At this magnification level, the particles appear as clustered aggregates composed primarily of smaller sub-particles. The agglomerations are generally smaller than about 1 µm and have quite irregular shapes.

The rough, wrinkled, and highly deformed surface topology of the particles is quite remarkable.

Images taken after oxidation at 650 °C/4 h Figure 3c,d show a significant change in morphology: in the 250× image Figure 3c, the particles now appear as finer, looser, and more dispersed small aggregates instead of compact clusters. In the 10,000× image Figure 3d, these fine particles form a loosely connected, branched/agglomerated (dendritic-like) network structure, but not as dense and compact as the clusters as in Figure 3b.

**Figure 4:**
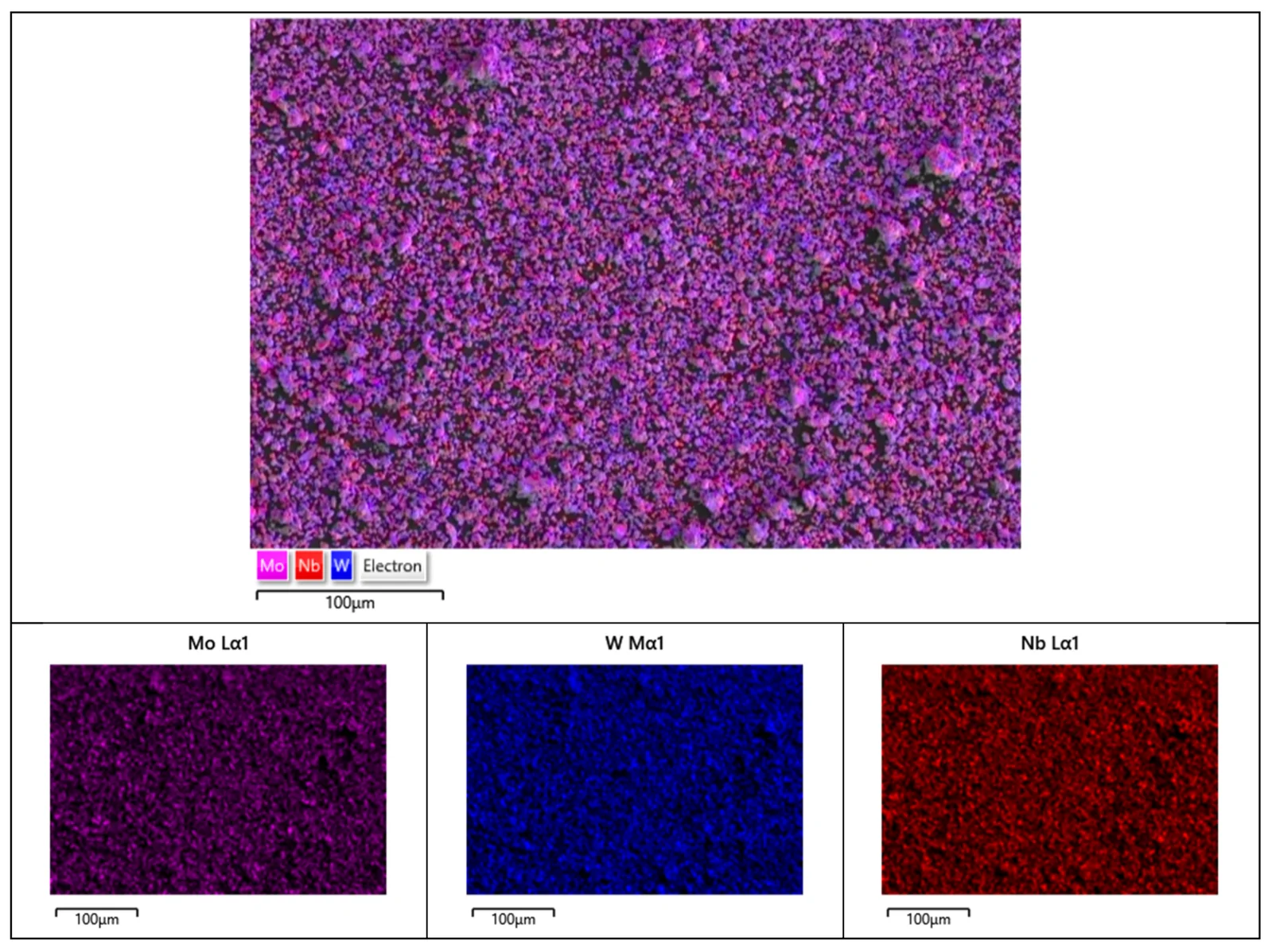
EDS mapping of 150 h mechanical alloyed WMoNb powder.

**Figure 5:**
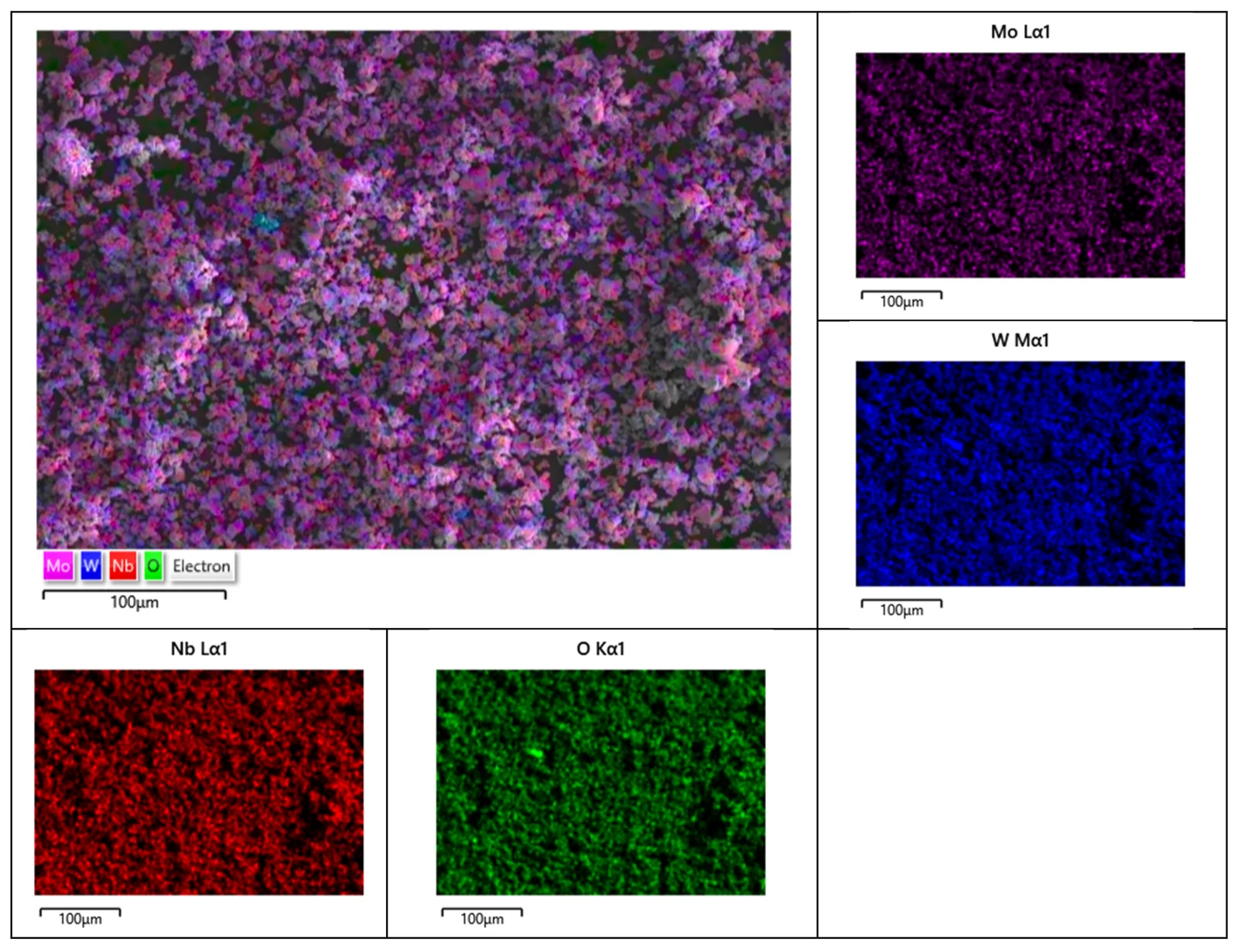
EDS Mapping of oxided at 650 °C/4-h after 150 h mechanical alloyed WMoNb powder.

EDS element mapping performed on WMoNb powder after 150 h of mechanical alloying, as shown in Figure 4, confirms that W, Mo, and Nb exhibit a homogeneous distribution throughout the studied region. Figure 5 shows the EDS analysis of RMEA oxide powder after 150 h MA.

Mapping analyses reveal that the signals of the three elements overlap uniformly across the entire particle cluster, with no element-rich or element-poor regions. The map obtained after oxidation shows that the individual elemental distributions of Mo, W, and Nb exhibit a homogeneity largely similar to the previous (unoxidized) state. The 650 °C/4 h heat treatment did not significantly alter the positional distribution of the metallic elements relative to each other; the three elements still remain widely distributed on the particles in similar proportions. In addition, the added “O” map shows that oxygen is distributed across the entire particle surface, not confined to a specific region.

### 3.2. Characterization of Composites

#### 3.2.1. EDS Analysis of Al Reinforced with RMEA

**Figure 6:**
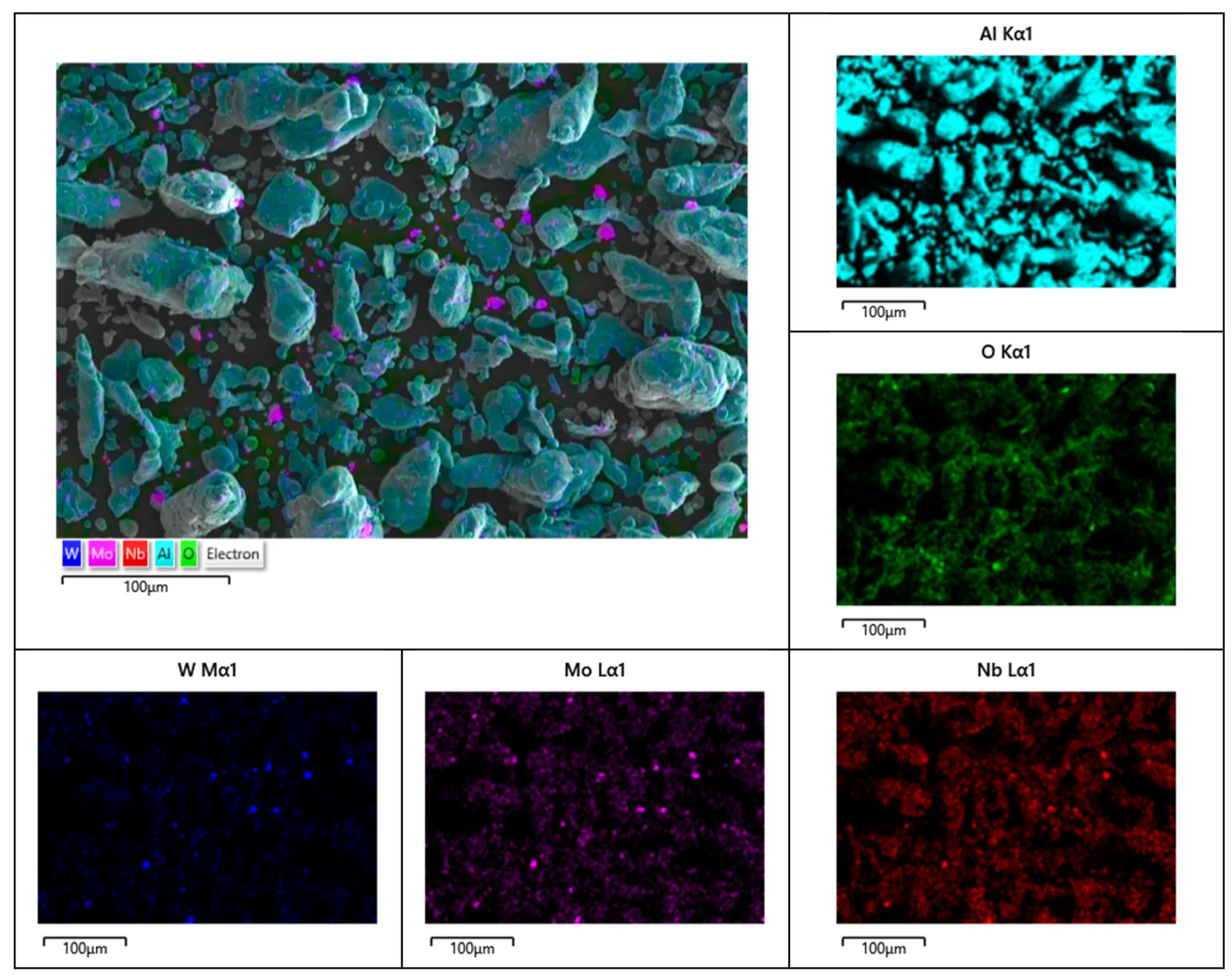
EDS mapping image of composite powder with 2.5 wt.% RMEA additive.

**Figure 7:**
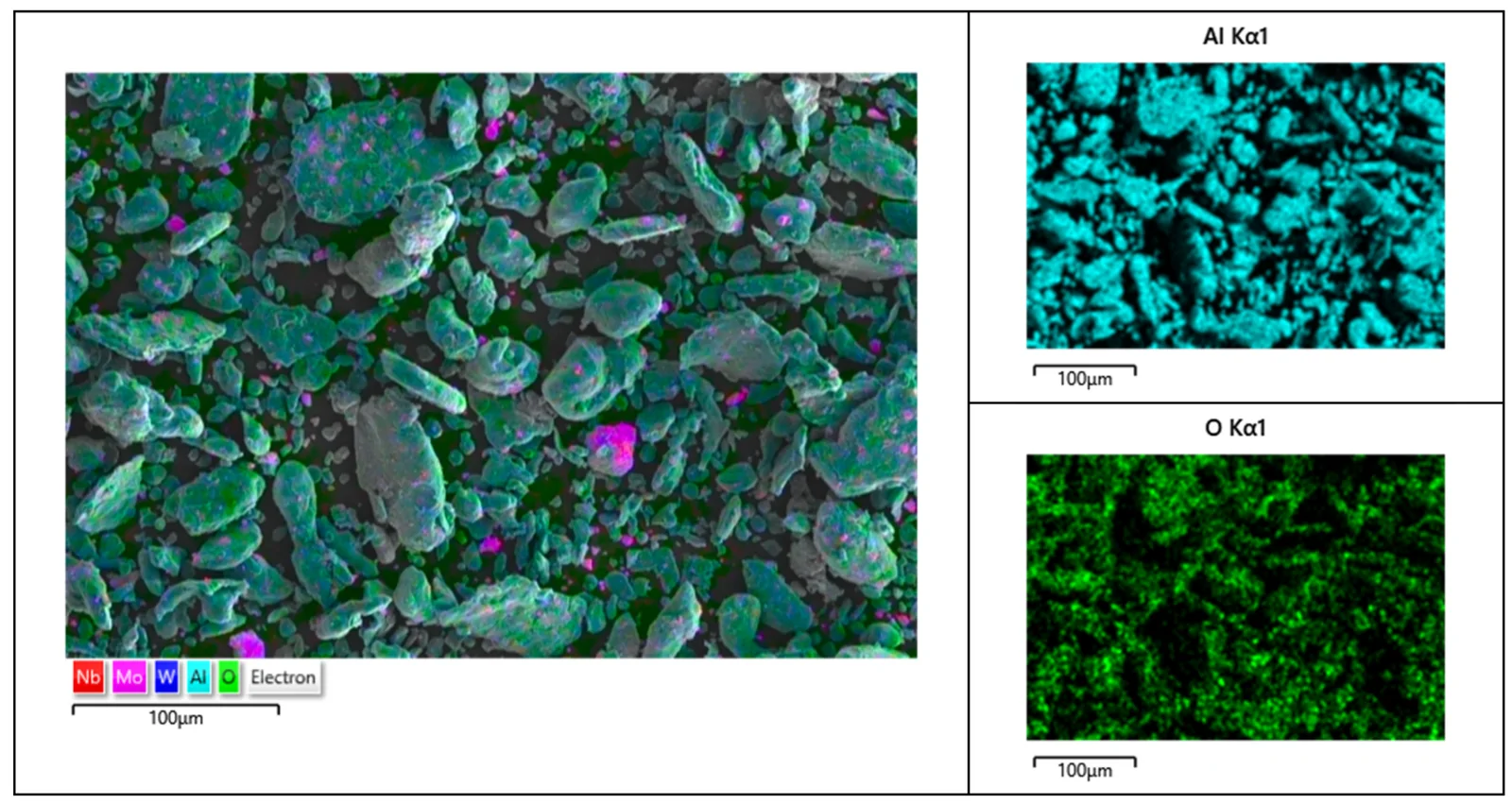

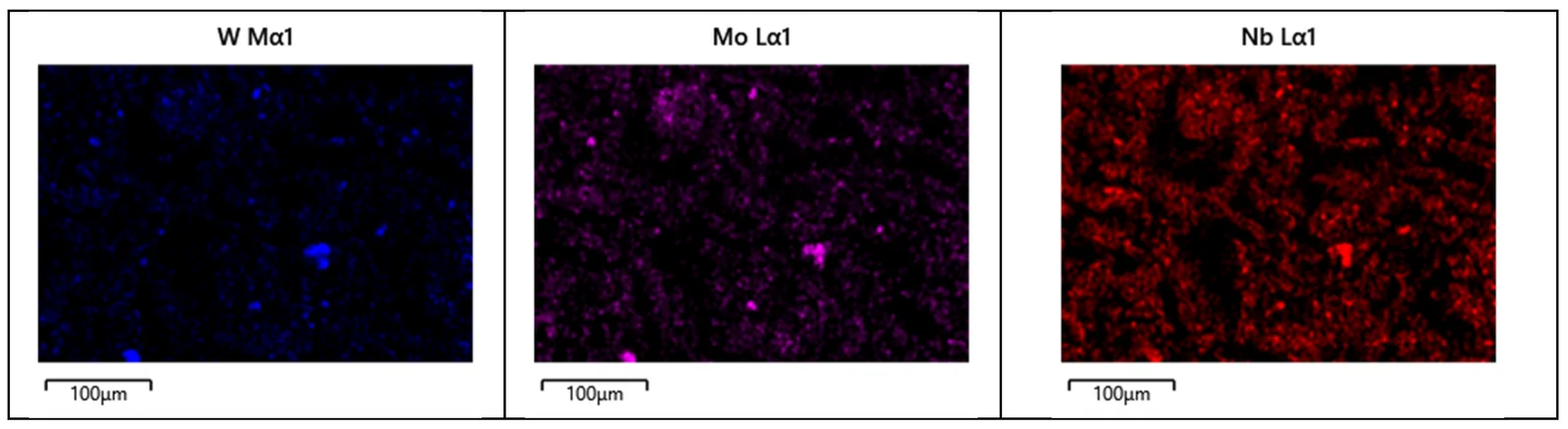
EDS mapping image of composite powder with 5 wt.% RMEA additive.

**Figure 8:**
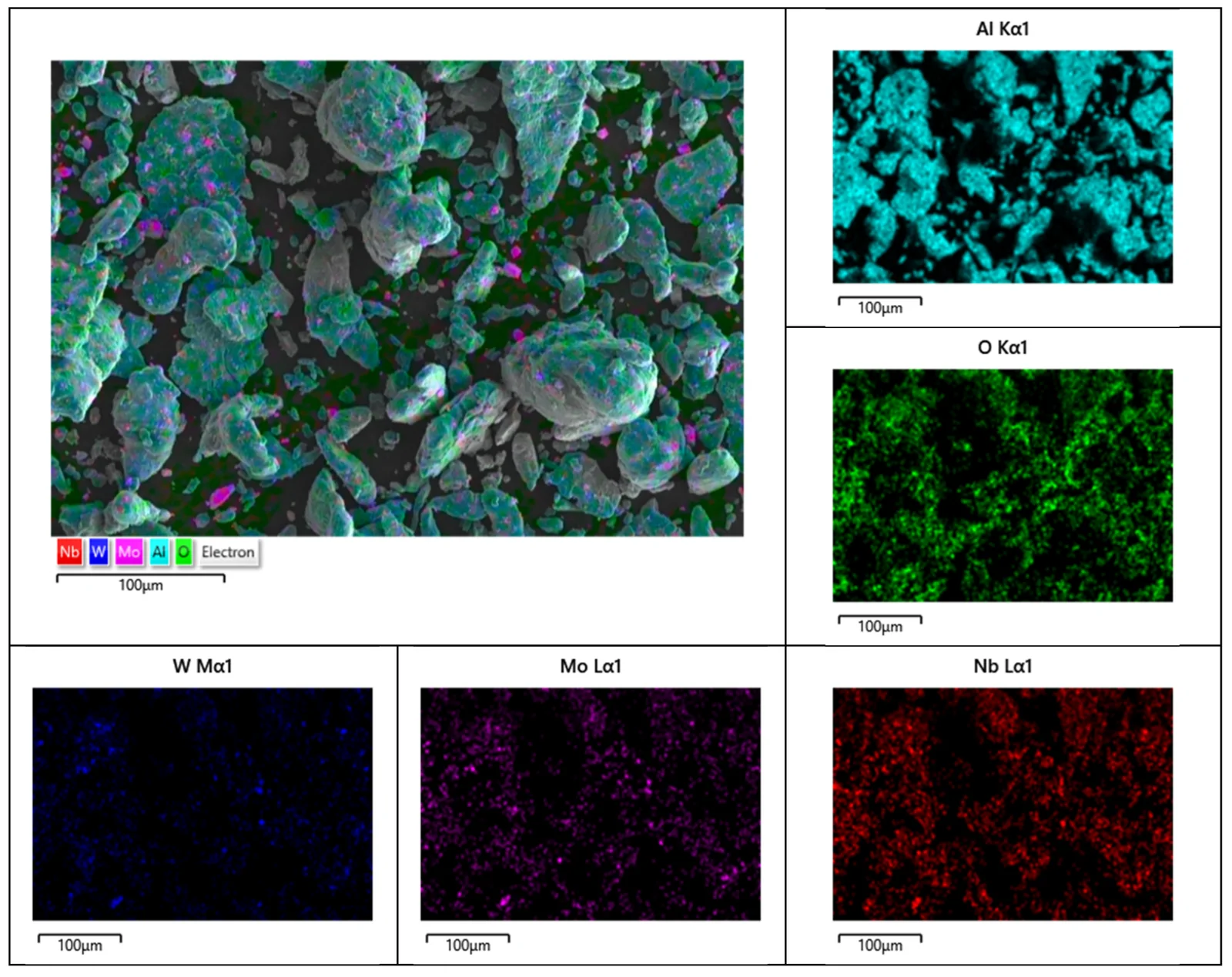
EDS mapping image of composite powder with 7.5 wt.% RMEA additive.

**Figure 9:**
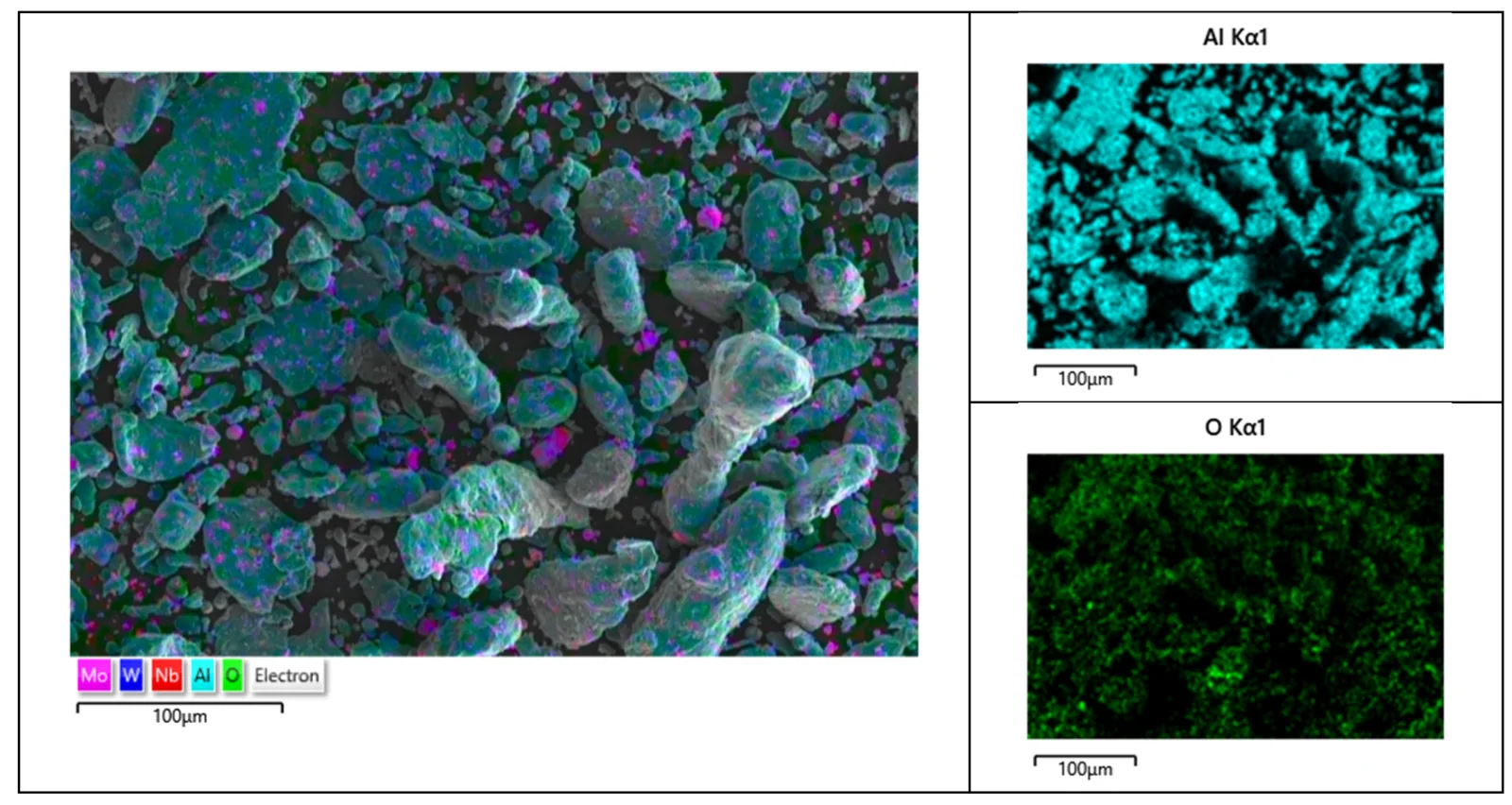

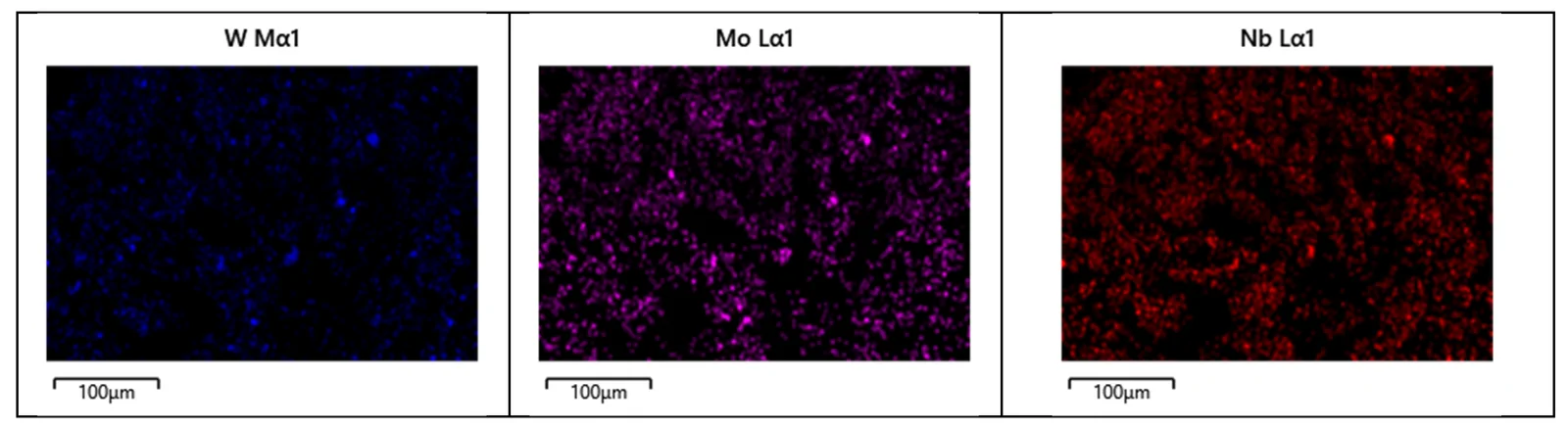
EDS mapping image of composite powder with 10 wt.% RMEA additive.

EDS mapping images of composite powders with 2.5%, 5%, 7.5%, and 10% RMEA additives are provided in Figure 6, Figure 7, Figure 8 and Figure 9, respectively. A systematic comparison of EDS mapping analysis obtained from Al-2.5, Al-5, Al-7.5, and Al-10 weight percentage WMoNb powder mixtures shows a clear and gradual transition in the distribution of refractory RMEA reinforcement depending on increasing WMoNb content, while the stability of the RMEA phase is visible in all images. In the Al-2.5 weight percentage WMoNb composite, W, Mo, and Nb appeared as sparsely distributed, completely isolated bright regions with low field density, wide interparticle distance, and no signs of aggregation, exhibiting the most homogeneous distribution among all mixtures studied. As the WMoNb content increased to 5%, although the number density of refractory element-rich regions in the element maps increased proportionally, the distribution remained acceptably homogeneous without macroscopic clustering, indicating that mixing conditions were equally effective at this percentage level. At a weighting of 7.5% WMoNb, a greater increase in the areal ratio of WMoNb-rich regions is observed, and local regions with increased interparticle proximity become more prominent in certain areas of the maps. Although a discontinuous WMoNb network rather than a complete connection is not detected, it can be interpreted as the beginning of a transition regime where higher reinforcement density begins to reduce the average separation distance between neighboring particles.

In the Al-10% WMoNb mixture, the W, Mo, and Nb maps reveal the highest density of refractory-rich regions, showing concentrated WMoNb particles in some areas and a locally reduced Al matrix.

**Figure 10:**
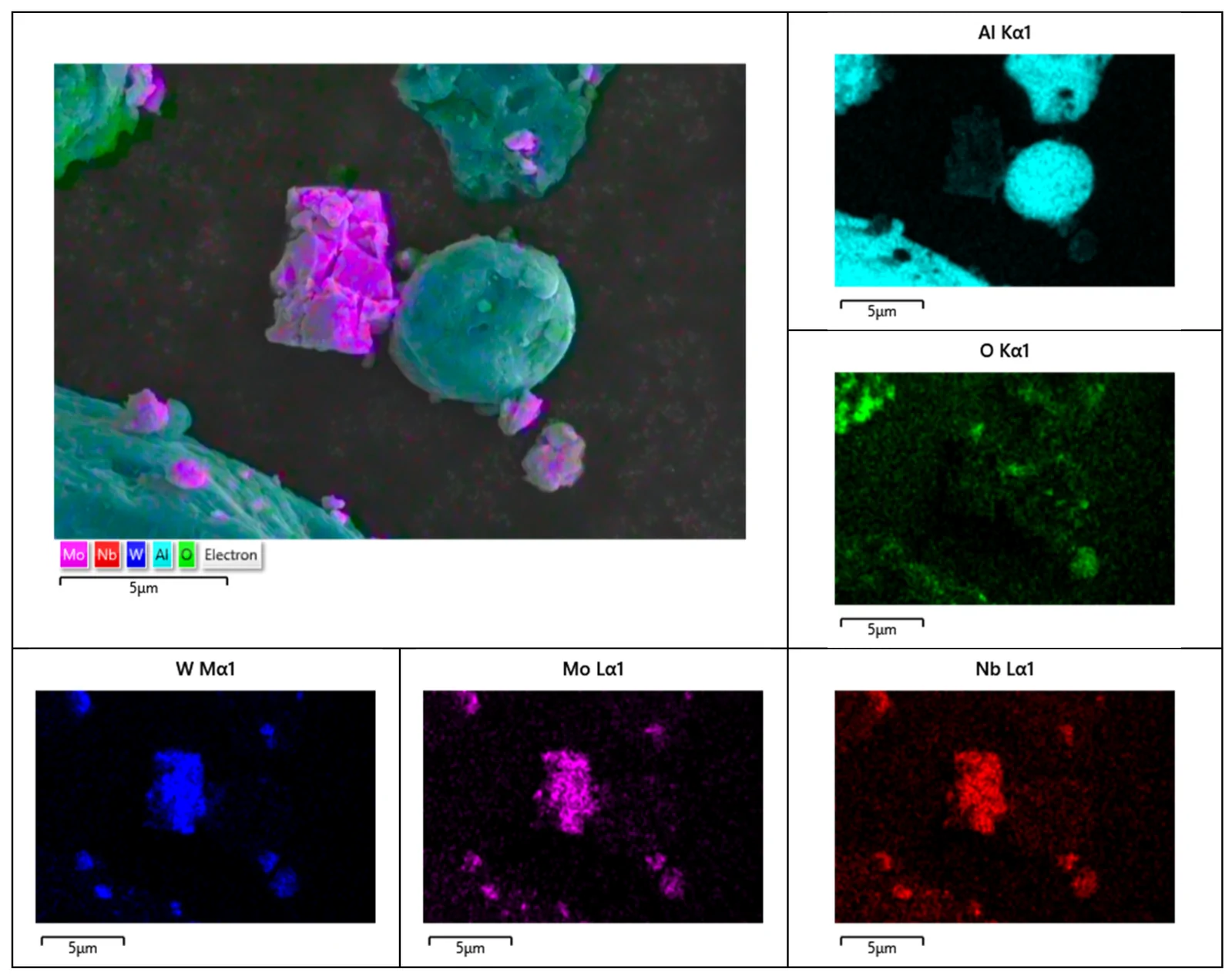
EDS mapping image of composite powder (RMEA and Al).

Although no evidence of oxygen was found in the structure related to WMoNb powder production, the presence of oxygen was observed in the EDS mapping analysis, in Figure 10. The maps of Al-O elements and W–Mo–Nb elements can be interpreted as two different maps that overlap with each other. 

Alongside the spherical (unprocessed) raw Al powder particle, i.e., in its natural state, a heavily deformed RMEA powder particle, resembling a rectangular prism after repeated fracture-fusion processing, is clearly visible. Cylindrical or spherical RMEA powder particles are also clearly visible in the image.

#### 3.2.2. EDS Analysis of Al Reinforced with Refractory Medium-Entropy Alloy Oxide (RMEO)

**Figure 11:**
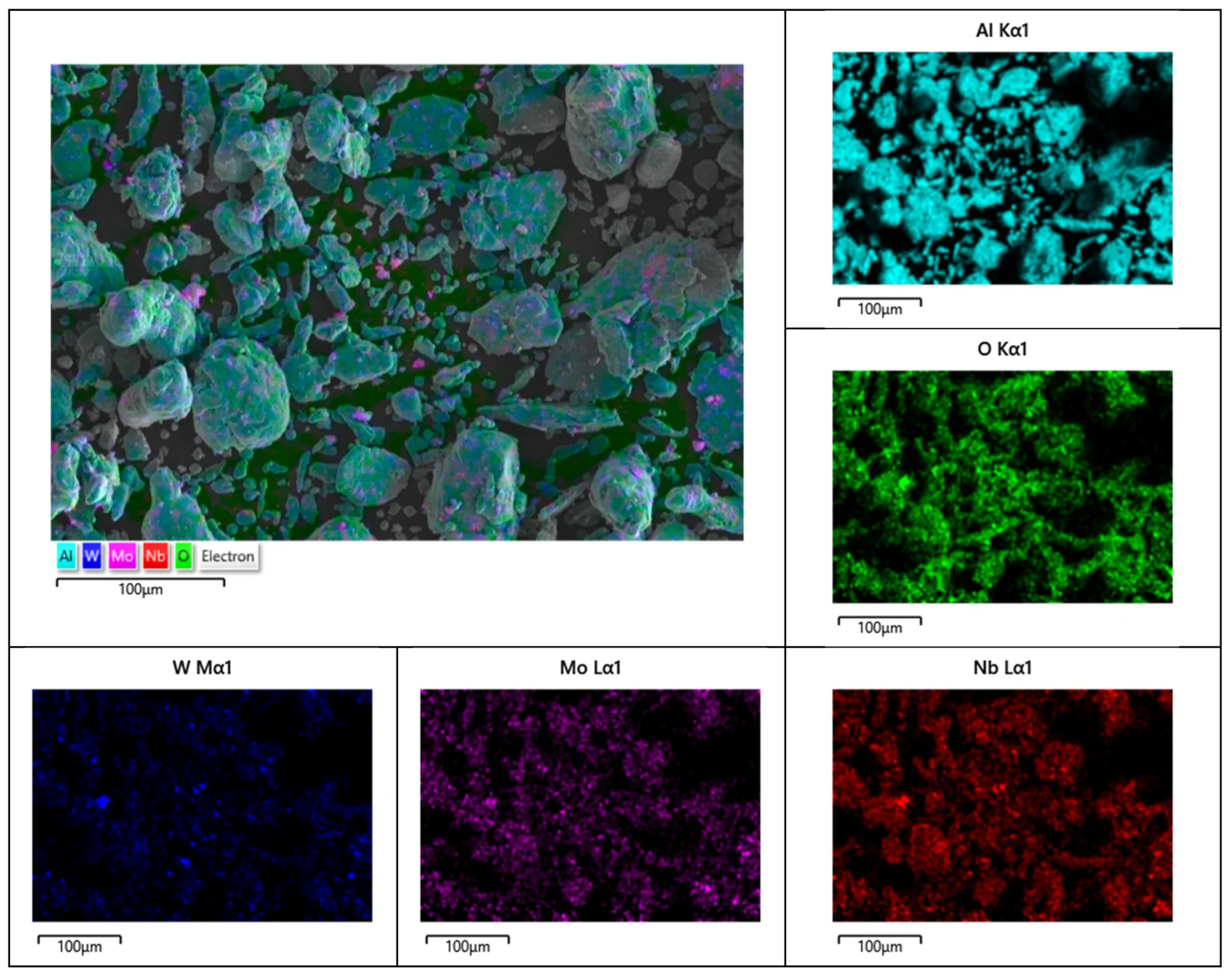
EDS mapping image of composite powder with 2.5 wt.% RMEO additive.

**Figure 12:**
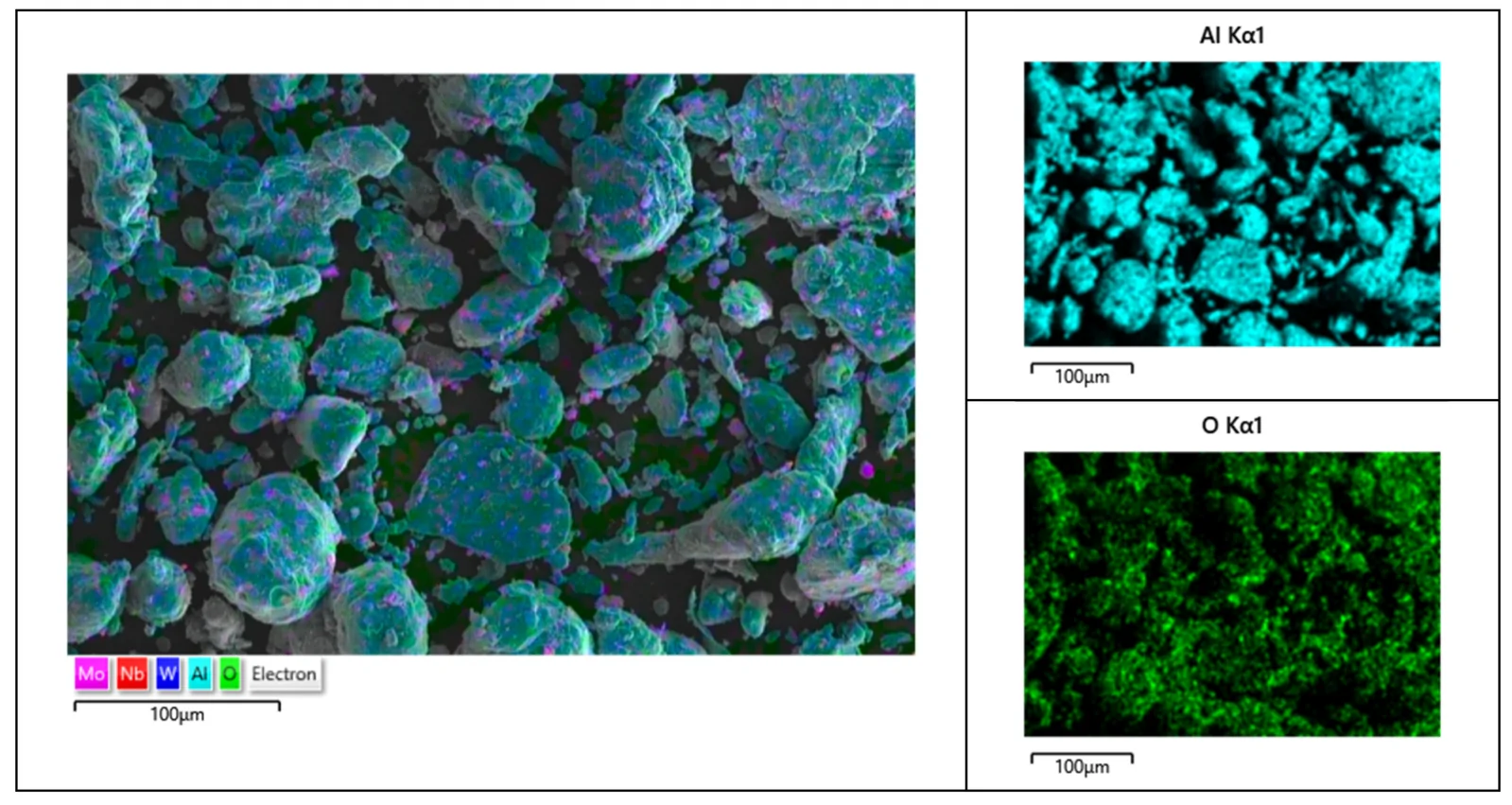

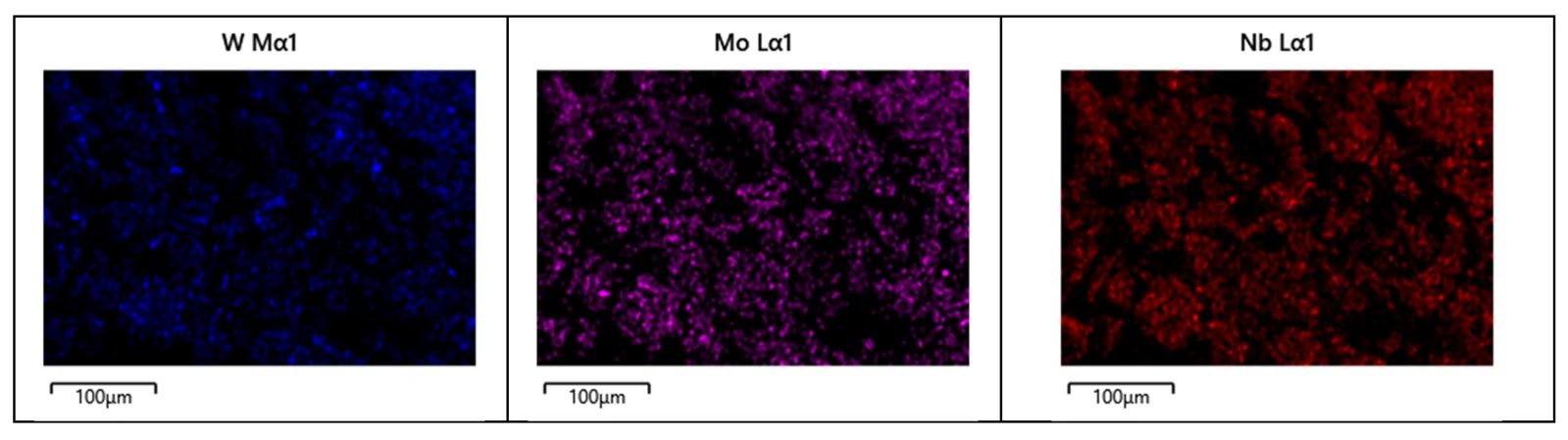
EDS mapping image of composite powder with 5 wt.% RMEO additive.

**Figure 13:**
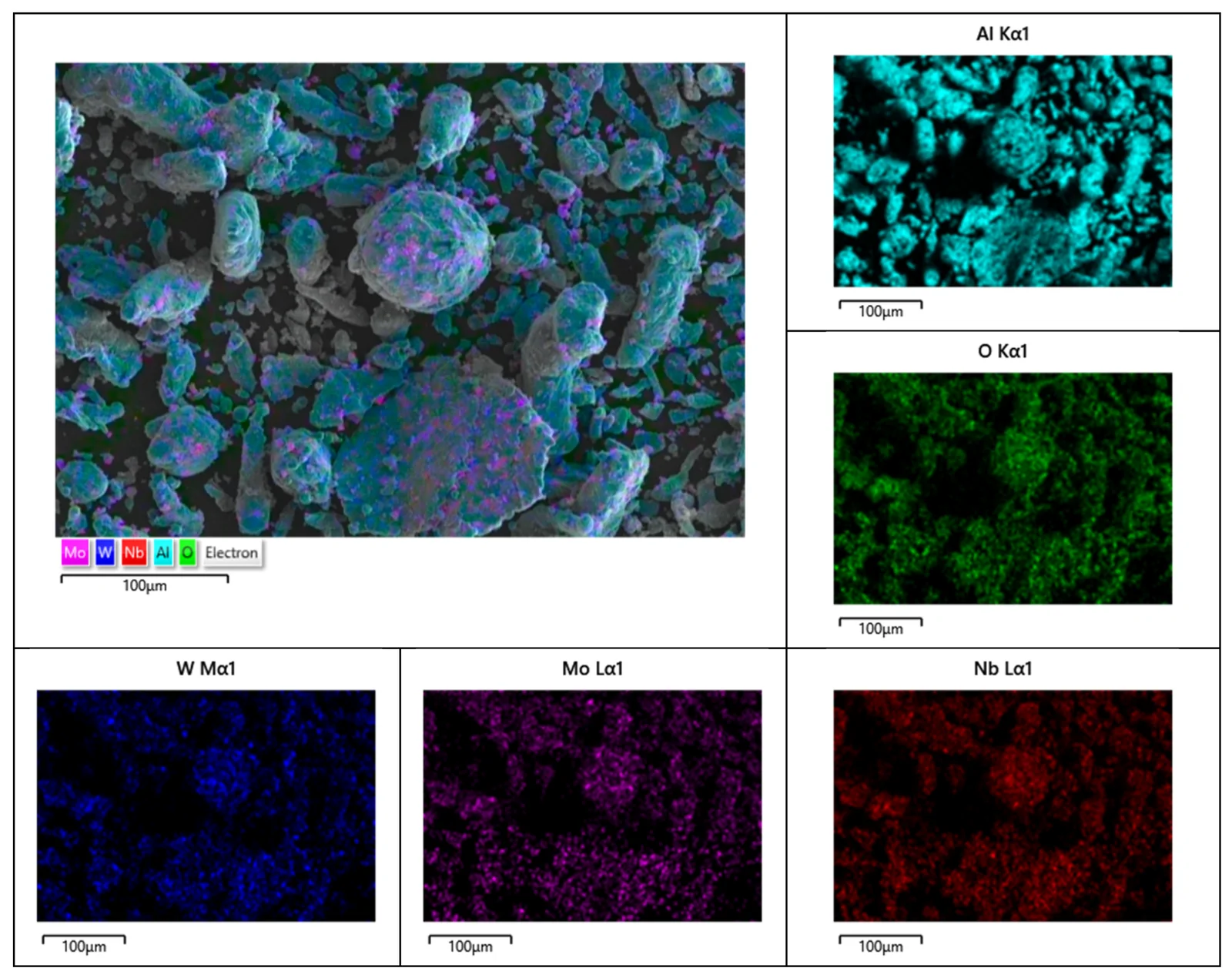
EDS mapping image of composite powder with 7.5 wt.% RMEO additive.

**Figure 14:**
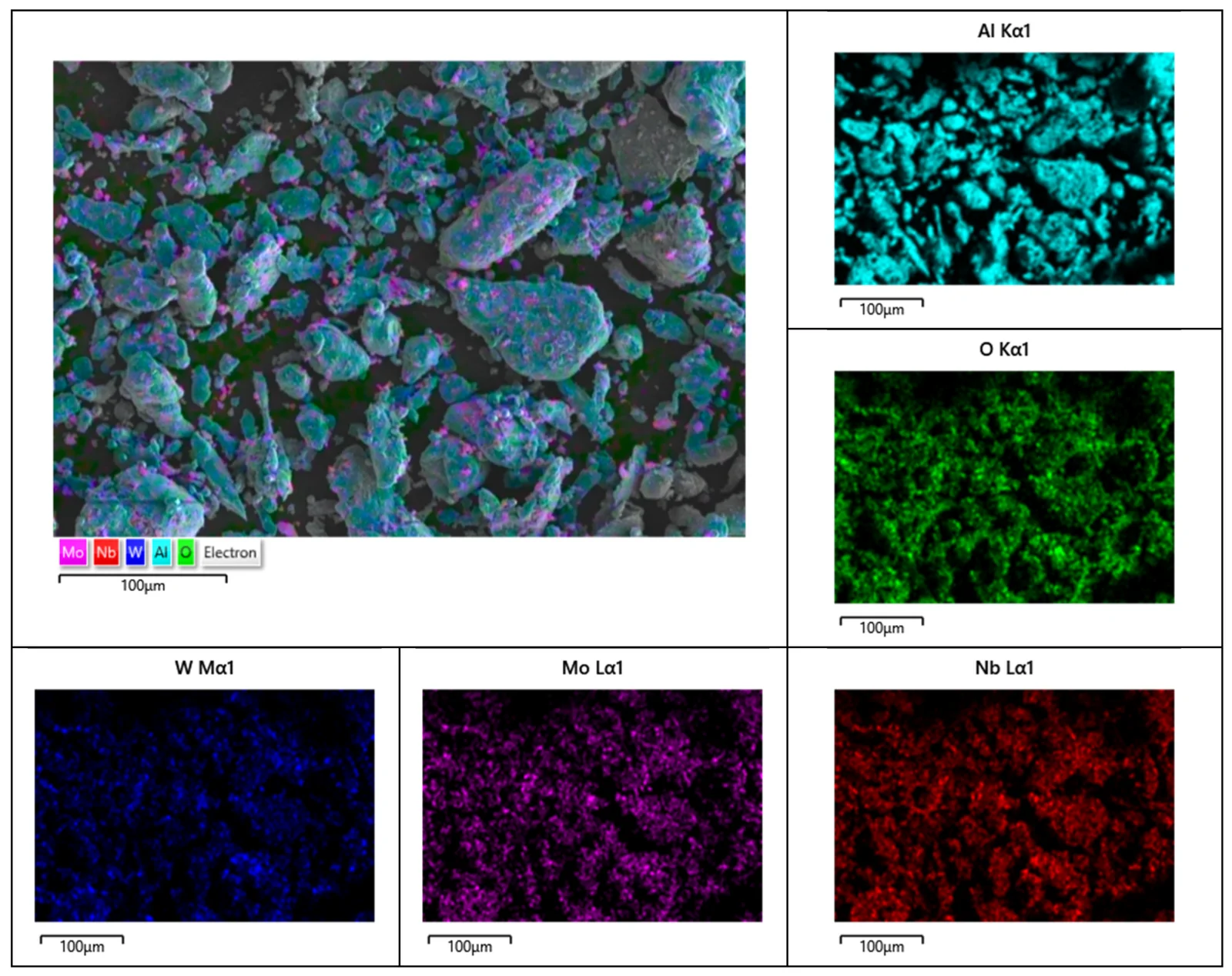
EDS mapping image of composite powder with 10 wt.% RMEO additive.

EDS mapping images of composite powders with 2.5%, 5%, 7.5%, and 10% RMEO additives are provided in Figure 11, Figure 12, Figure 13 and Figure 14, respectively.

In Figure 11, EDS mapping shows that the low-percentage (2.5%) RMEO reinforcement is located in a relatively fine and dispersed manner in the intergranular regions within the Al matrix powder, without showing agglomeration. The Al matrix is clearly distinguishable as the coarse-grained, dominant phase in the EDS map, and the MEO reinforcing elements (W, Mo, Nb) are also clearly seen to be present in low proportions and without clustering, scattered in the intergranular regions.

In Figure 12, EDS mapping shows that increasing the reinforcement ratio from 2.5% to 5% significantly increases the density and distribution frequency of W, Mo, and Nb in the elemental image area. However, a denser oxygen (O) coat develops around and on the surface of the Al grains.

Figure 13 shows that EDS mapping significantly increases the elemental density and areal coverage of Mo and Nb compared to previous compositions (2.5% and 5%) when the RMEO reinforcement ratio is increased to 7.5%, and that oxygen distribution also forms a more widespread network among and on the surfaces of Al grains.

Figure 14 shows that increasing the RMEO reinforcement ratio to 10% results in Mo, Nb, and W being distributed across the entire image area in an almost continuous and dense texture, while oxygen distribution expands to the point where it forms a distinct, network-like covering on the surface of most Al grains; this represents the densest reinforcement and highest oxide load among the four compositions studied (2.5%, 5%, 7.5%, 10%).

### 3.3. Wear Test Analysis

**Figure 15:**
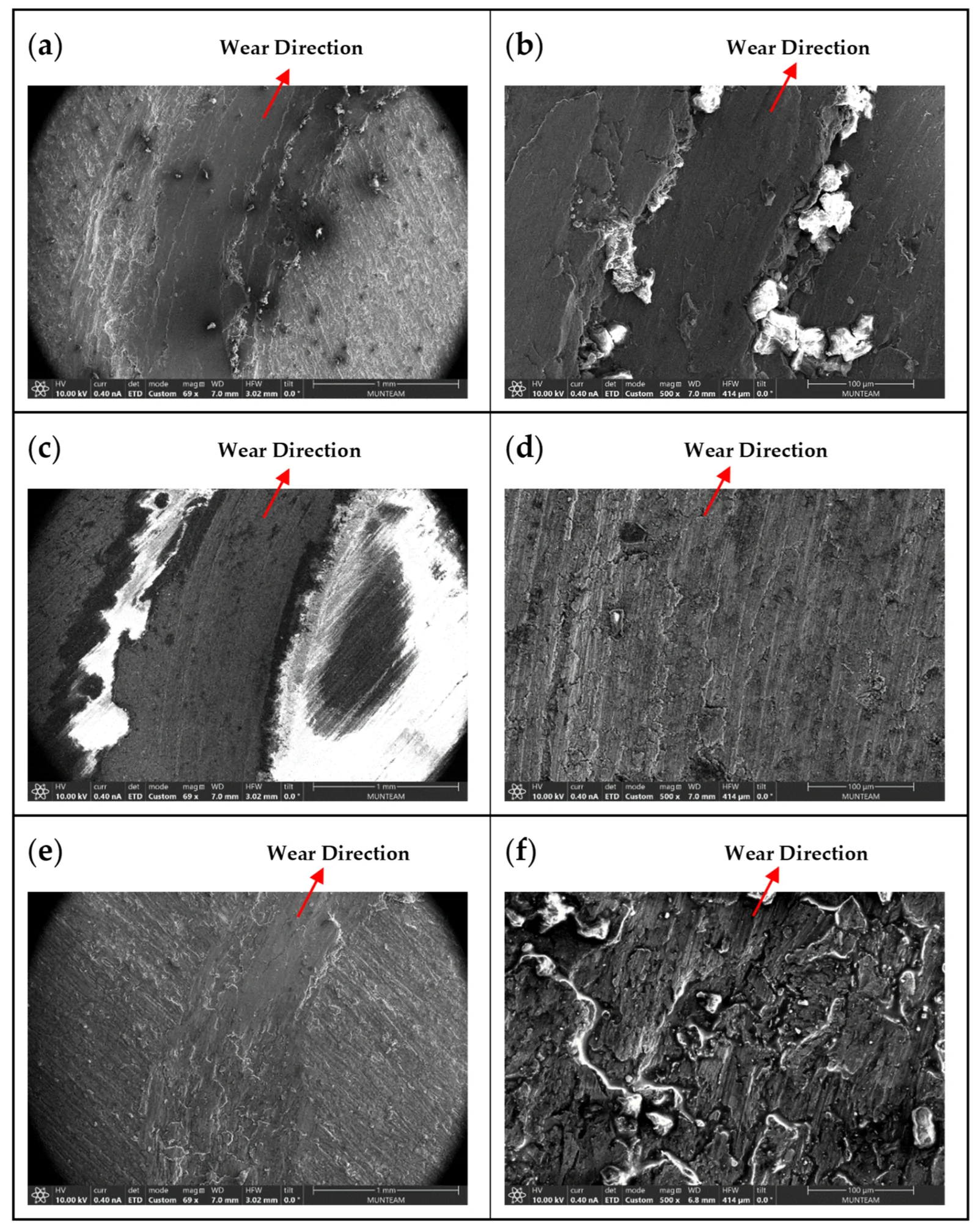
(**a**,**b**) SEM images of the wear surface of pure aluminum. (**c**,**d**) Images of an aluminum matrix composite reinforced with 10 wt.% RMEA. (**e**,**f**) Images of an aluminum matrix composite reinforced with 10 wt.% RMEO, at 69× and 500× magnification, respectively.

Wear surface images of pure Al, reinforced with 10 wt.% RMEA and reinforced with 10 wt.% RMEO with different magnifications, are given in Figure 15. Parallel linear marks (scratch marks/grooves) perpendicular to the direction of friction are clearly visible at all magnifications.

In the 500× magnification image (Figure 15b), white, shiny, irregularly morphological debris clumps are noticeable at the edges of the wear tracks. In Figure 15d, the interlocking network of cracks concentrated in the left half of the image is a typological indicator of fatigue-induced delamination, which occurs when the plastic deformation energy accumulated on the underlying surface exceeds a critical threshold. In the 69× image (Figure 15e), the surface consists of fine, regular, shear-directional scratches, similar to those in pure Al Figure 15a, but large, bright adhesion areas like those in Figure 15c are not visible.

**Figure 16:**
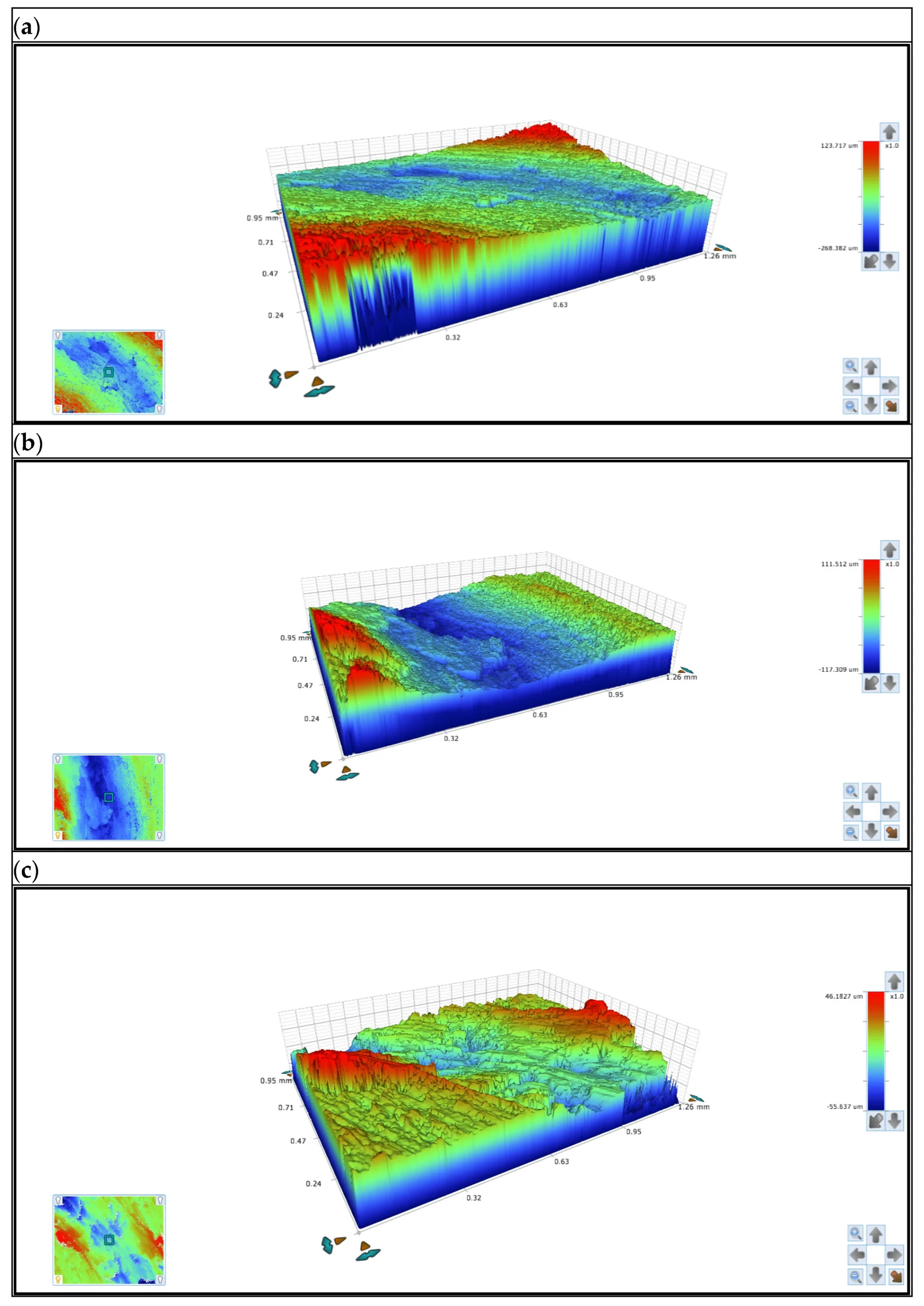
3D profilometer analysis of (**a**) pure Al, (**b**) 10 wt.% RMEA–Al, and (**c**) 10 wt.% RMEO–Al samples.

In Figure 16, 3D profilometer analysis of Figure 16a pure Al, Figure 16b 10 wt.% RMEA–Al, and Figure 16c 10 wt.% RMEO–Al samples is given.

In Figure 16a, the color scale ranges from −268.382 µm (blue/deep) to +123.717 µm (red/high), with a total height range of approximately R_t_ ≈ 392 µm. In the wear channel image, the dark blue regions extending along the mid-left axis represent the area of most intense tangential shear contact; the wear depth was measured as h_max_ ≈ −268 μm.

In Figure 16b, 3D profilometer analysis of 10 wt.% RMEA is given. The color scale ranges from −117.309 µm (blue) to +111.512 µm (red), with a total height range of R_t_ ≈ 229 μm. The h_pile-up_ distance was calculated as +111.5 μm by measuring the distance between point 0 and the minimum wear depth on the scale in Figure 16b.

Figure 16c shows the 3D profilometer analysis of the wear track of the 10 wt.% RMEO-reinforced Al matrix composite. The total height difference of the surface was measured as R_t_ ≈ 101.8 μm (between +46.183 μm and −55.637 μm).

Table 3 presents the 3D profilometer analysis data of the samples. Reduction factor vs. pure Al values were calculated using the ratios of both RMEA and RMEO h_max_ to the h_max_ of pure aluminum.

When the three 3D profilometer analyses are considered together, it is clearly seen that the wear track depth and edge pile-up systematically decrease as one progresses from unreinforced pure Al to 10 wt.% RMEA-reinforced composite and finally to 10 wt.% RMEO-reinforced composite, thus showing a gradual increase in wear resistance. In the RMEO sample, the wear track depth was reduced by approximately four times compared to pure Al and by more than two times compared to RMEA.

**Figure 17:**
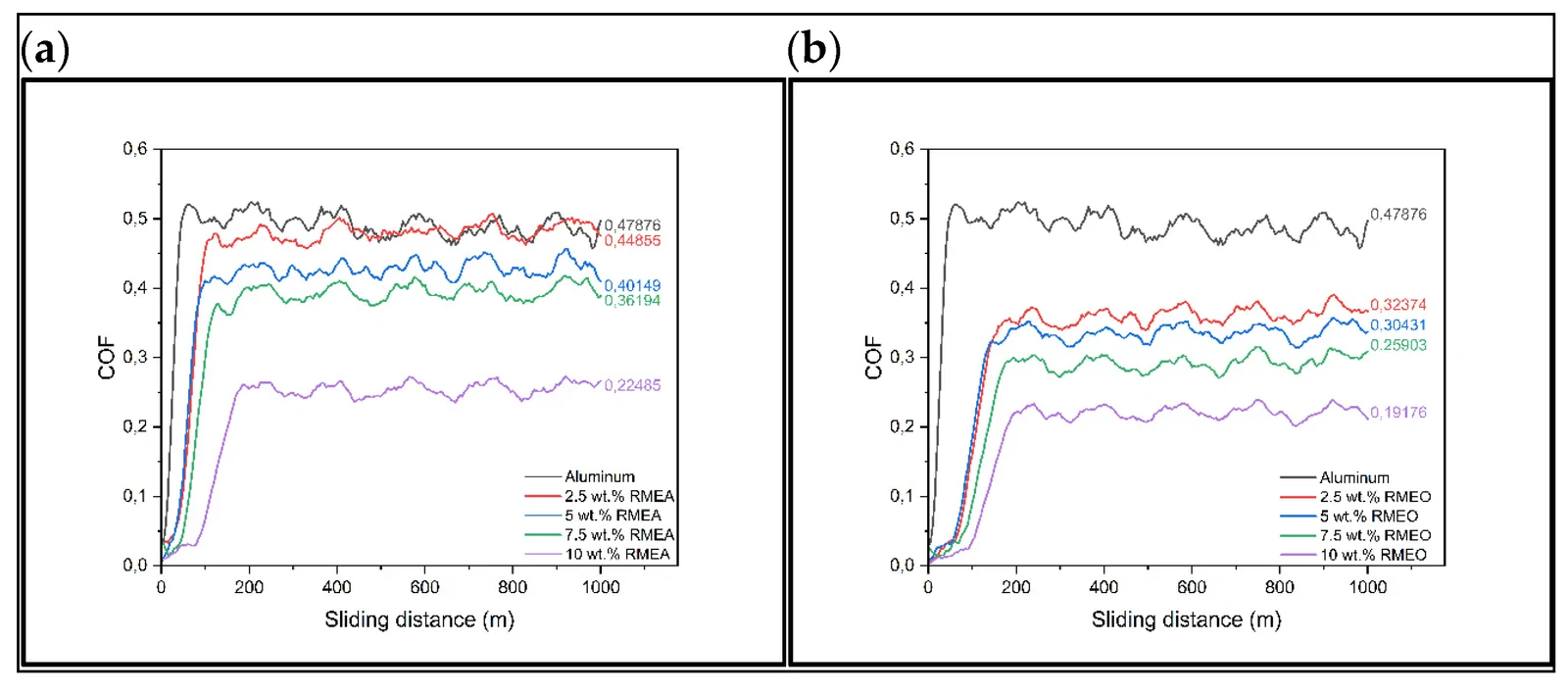
COF–sliding distance graph of (**a**) Al–RMEA and (**b**) Al–RMEO.

In the Al–RMEA and Al–RMEO wear comparison (Figure 17), when looking at the COF–sliding distance graph, all samples exhibited a rapid transient increase in their COF in the first 0–100 m range of the sliding distance, followed by a stabilization.

The pure aluminum sample exhibited the highest peak COF with a μ_peak_ value of ≈0.52, reaching this value within approximately 50 m.

Average COF values, wear loss, hardness in the steady-state region, and percentage decreases compared to the pure Al reference are given in Table 4. When Table 4 is examined in detail, it can be seen that the hardness values increase significantly with increasing reinforcement ratio, rising from 61 HB for pure Al to 114 HB for 10% RMEA and to 132 HB for 10% RMEO. RMEO samples exhibit higher hardness than RMEA at all ratios, meaning that a hard oxide/particle dispersion effect is created within the structure [26].

**Figure 18:**
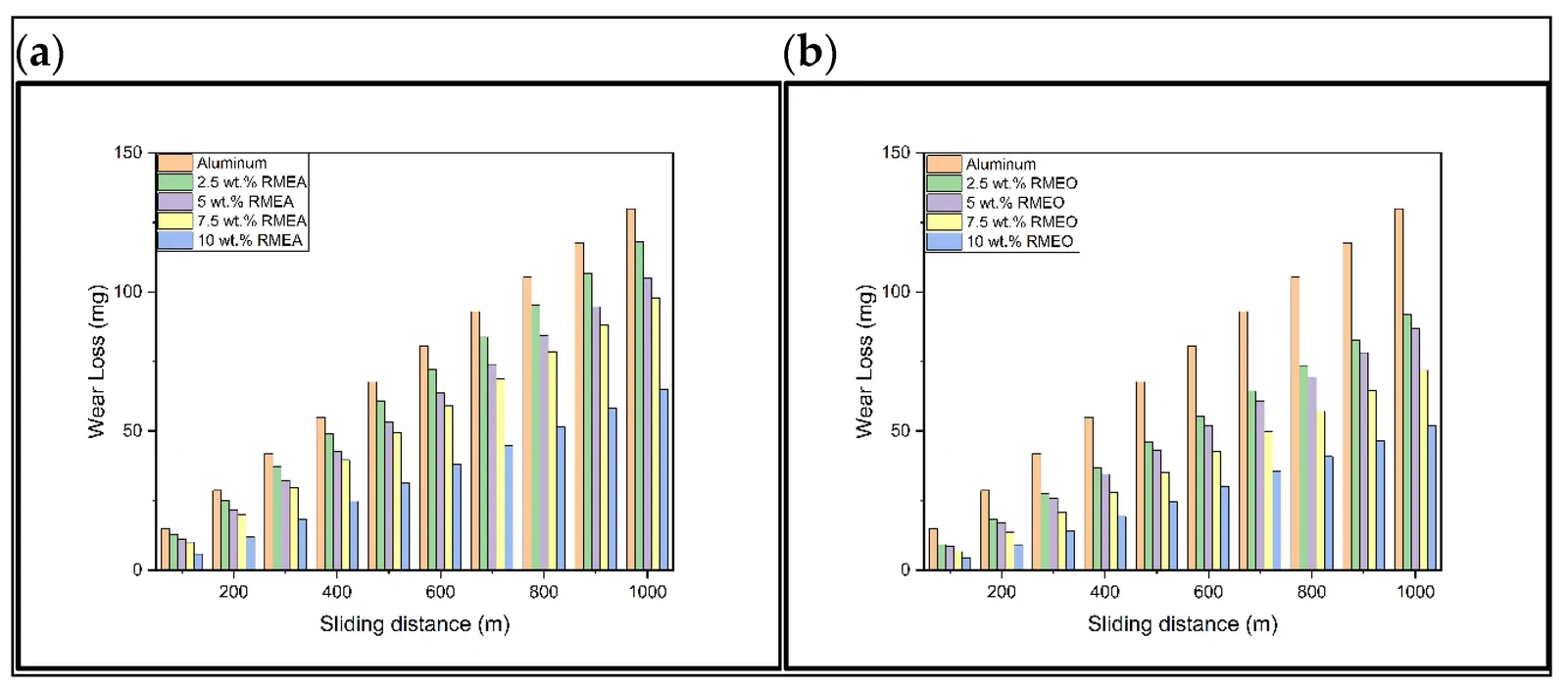
Wear loss–sliding distance graph of the (**a**) RMEA and (**b**) RMEO samples.

Figure 18 shows the wear loss as a function of sliding distance for composites reinforced with pure Al and Figure 18a 2.5%, 5%, 7.5%, and 10% by weight of RMEA, and Figure 18b 2.5%, 5%, 7.5%, and 10% by weight of RMEO particles. It is evident that the tests were predominantly performed in the steady-state wear regime and are consistent with the Archard framework, where cumulative material loss scales proportionally with sliding distance, as the wear loss increases almost linearly with sliding distances between 100 and 1000 m. At a distance of 1000 m, pure Al showed the highest wear loss (≈130 mg), while the 10% by weight RMEA and 10% by weight RMEO composites showed the lowest values (≈65 mg and ≈52 mg, respectively), which corresponds to a reduction of approximately 50% and 60% compared to unreinforced Al.

## 4. Discussion

The formation of a single-phase BCC solid solution without secondary phases confirms that the prolonged high-energy milling process successfully promoted solid solution formation while suppressing undesired phase transformations [27].

The formation of a single BCC solid solution in the W–Mo–Nb ternary system is thermodynamically consistent with the high configurational mixing entropy of the equiatomic composition. According to Hume-Rothery rules, all three specified elements share the same BCC crystals and exhibit co-existing atomic fractions (r_W_ = 1.37 Å, r_Mo_ = 1.36 Å, r_Nb_ = 1.43 Å) and electronegativity values, which collectively support the reduction in solute content in the solid.

This peak broadening can be attributed to two concurrent phenomena:

*(i)*  A significant reduction in mean crystallite size resulting from the severe plastic deformation imposed by high-energy ball milling;
*(ii)*  The accumulation of non-uniform lattice microstrain caused by the incorporation of solute atoms with differing atomic ratios into the BCC lattice.

The above are consistent with reported findings for similar refractory HEA systems produced by HEBM.

This is a typical result of prolonged (150 h) high-energy ball milling. Such a long MA time creates significant lattice strain, grain reduction, and partial amorphization in the crystal lattice, leading to broadening of the Bragg peaks and increased background intensity, precisely the behavior observed in Figure 1. Similar WMoNbTaV and WNbMoVAlCr refractory HEA/MEA powders have also been reported in the literature, showing the disappearance of elemental peaks and a contraction–expansion equilibrium towards a single BCC phase at long MA times, but with extremely long milling times, a nanocrystalline/amorphous character becomes prominent [28,29]. It can be said that after heat treatment, the powder does not transform into a single homogeneous oxide phase, but instead exhibits an oxidation behavior that differs on an elemental basis [30]. From the perspective of W, it can be observed that the lines corresponding to the peak positions of the main BCC tungsten phase are maintained under a relatively mild oxidation condition such as 650 °C/4 h. This is consistent with the information that W oxidizes more slowly than other elements and generally transforms into WO_3_ at a higher temperature/after a longer time. The fact that Mo is more reactive to oxygen than W explains why its partial oxidation occurs before W at this temperature. The absence of the complete oxide of Mo (from Mo to MoO_3_) means that its oxidation is incomplete. Similarly, the transformation of Nb into an oxygen-deficient sub-oxide (NbO_x_, x < 1) phase instead of complete Nb_2_O_5_ indicates that the oxidation kinetics of Nb are also incomplete, in an intermediate stage. These results show that WMoNb powder undergoes selective oxidation at 650 °C, meaning that the three elements do not oxidize at the same rate: Nb and Mo are primarily oxidized because they have a higher thermodynamic affinity for oxygen compared to W, while W largely retains its metallic BCC structure. This behavior is consistent with the phenomenon of selective oxidation frequently reported in refractory HEA/RHEA systems; for example, in Ta-containing WMoNbTaV coatings, it has been reported that Ta-rich oxides are primarily formed due to Ta’s high affinity for oxygen, while the other elements remain relatively metallic [30].

This morphology is typical for powders subjected to prolonged high-energy ball milling, where repeated cold welding and fracture events eventually reach a dynamic equilibrium [26].

Nevertheless, the overall particle distribution can be considered relatively homogeneous, suggesting that the 150 h milling treatment successfully refined the powder and reduced the extent of coarse, unmilled particles.

In many regions, the particles exhibit layered or lamellar-like surface features characteristic of repeated cold welding and plastic deformation processes occurring during HEBM [31]. These observations suggest that the powder particles underwent extensive flattening, re-welding, and subsequent fracture during milling, producing a highly corrugated surface morphology.

The absence of smooth crystallographic surfaces and the presence of significant surface distortion can be considered an indication that the particles contain a high density of crystal defects such as dislocations, grain boundaries, and tension fields. This observation is in good agreement with the XRD peak broadening, which suggested substantial crystallite refinement, as shown in Table 1. Therefore, SEM and XRD findings consistently demonstrate that WMoNb powder exhibits a nanocrystalline or highly defective microstructure after 150 h of MA.

In fact, from a composite perspective, rough and irregular surface morphology can be beneficial for the fabrication of Al matrix composites. Such surfaces can improve mechanical interlocking between the reinforcing particles and the metallic matrix, strengthening the particle–matrix interface. A strong interface is important for load transfer efficiency and wear resistance, especially in tribological applications.

Taken as a whole, XRD and SEM analyses provide a consistent picture of the structural evolution of WMoNb powder during long-term mechanical alloying (MA). XRD confirmed the formation of a single BCC solid solution phase with broadened diffraction peaks, indicating crystallite thinning and lattice disruption, while SEM revealed the external morphology associated with this structural state, namely fine particles, partial aggregation, and densely deformed rough surfaces. These findings are mutually supportive. The formation of a BCC solid solution explains the absence of visible phase segregation, while the severe surface deformation observed by SEM reflects the intense mechanical energy input required to achieve alloying at the atomic level.

This morphological change indicates that oxidation shifted the mechanical behavior of the material from ductile to brittle; the formation of oxide phases (Mo_4_O_11_, NbO_0.76_) greatly reduced the cold welding capacity of the particles while increasing their tendency to fracture and disintegrate. Furthermore, at the selected temperature, reaction with oxygen led to the nucleation of thin oxide layers/grains on the particle surface, producing a porous microstructure composed of smaller, weakly bound subunits; this is consistent with the increase in surface roughness and grain refinement typically observed in the oxidation of metal powders [32].

When these four SEM images (Figure 3) are evaluated together, it can be said that 150 h of MA produced a metallic/ductile structure in the W–Mo–Nb powder, forming compact, dense, and relatively large aggregates, while oxidation transformed this structure into a finer, more brittle, and loosely bound oxide-dominant morphology. This behavior directly coincides with the previous XRD data. The morphological refinement and embrittlement here complement each other, given that the metallic BCC phase (especially in terms of W) is partially preserved, but Mo and Nb are oxidized and separated into distinct phases.

The uniform overlap of W, Mo, and Nb elements across the particle cluster indicates that the initially separate element powders have been successfully alloyed at the microscale. Specifically, no W-rich or Nb-rich cores or Mo-segregated shells can be detected in Figure 4; this indicates that repeated cold welding and fracture during high-energy ball milling provide sufficient parameters for the mixing of the elements. The absence of distinct oxygen-rich regions on the maps also indicates that the measures taken to minimize oxygen during grinding were appropriate and effective. These EDS results are consistent with XRD data showing a single BCC solid solution phase without detectable secondary intermetallic or oxide phases, confirming that WMoNb powder can be considered a compositionally homogeneous RMEA. The widespread distribution of oxygen (in Figure 5) across the entire particle surface indicates that the oxidation occurred as a simultaneous and widespread surface reaction throughout the entire particle population, not just in a specific part of the powder. When Figure 4 and Figure 5 are read together, it can be concluded that the MA process primarily provides complete homogenization at the metallic level, while the oxidation process adds an additional oxygen component to this homogeneous metallic skeleton without disrupting it; that is, oxidation is a secondary reaction that superimposes on the existing homogeneous structure, rather than rearranging the elemental distribution. The formation of a widespread but relatively limited layer of oxygen on the particle surface is consistent with the claim that the metallic W–Mo–Nb core is preserved and only a thin Mo/Nb-oxide-dominant shell develops on the surface.

On the other hand, EDS mapping analyses of Al matrix composites show that the highest refractory-rich regions and locally decreased Al matrix density observed at 10% WMoNb indicate that the refractory RMEA phase begins to restrict the continuity of the Al matrix at the highest reinforcement percentage level. However, the absence of macroscopic clustering is a good indication that the mixing process retains its effectiveness even at this upper reinforcement limit. The strong coexistence of W, Mo, and Nb in the same microstructural regions across all four reinforcement ratios clearly demonstrates that the homogeneity of the WMoNb RMEA phase formed during 150 h of high-energy mechanical alloying was fully preserved after mixing with Al powder, that there was no selective enrichment or reduction of individual refractory elements, and that no detectable oxygen-related problems were observed in any of the mixtures (related to WMoNb powder production). In short, EDS mapping analyses examining the mechanical alloying and powder mixing procedure collectively demonstrate that it creates a homogeneous distribution and a repeatable production path for Al–WMoNb composite powder systems across the entire reinforcement range studied (2.5–10% by weight). This is due to the presence of oxygen penetrating the structure because of impurities in the Al powder; this explains the reason for the overlap of oxygen with Al. The fact that WMoNb produced by the 150 h MA process remains homogeneously distributed and does not separate even after being mixed with Al powder, in other words, that no redistribution or separation of individual refractory elements is detected, shows that the mixing process does not compromise the integrity of the RMEA particles. Figure 10 illustrates this situation very clearly. EDS elemental maps of the mixed powder containing spherical Al particles and WMoNb MEA reinforcement (Figure 10) provide direct evidence of the morphological and chemical properties of the two-phase powder mixture before pressing. Oxygen, which was not detected in the EDS mapping after the MA process, was detected after mixing with Al, and it was suggested that the source of the trace amounts of oxygen in the structure was due to Al impurity. 

The linear morphology observed in the wear tests of the samples is a typical example of two-body abrasive wear, where hard particles on the surface or roughness from the opposite surface are embedded in the matrix, scratching the material. Low hardness of pure aluminum results in poor resistance to this mechanism. Structures represent adhesive tear residues (adhesive debris/transfer layer) that are either detached from the opposite surface and transferred or formed as a result of local plastic deformation. The high ductility and low yield strength of pure aluminum make adhesive wear prominent. The literature reports that adhesive wear is similarly the dominant mechanism in aluminum-based materials produced by powder metallurgy [33]. At the same magnification, thin layers of peeling and delamination marks are observed on the surface within the wear track; particularly, there are layered separation areas at the edges of the wear track. This indicates that repetitive contact stresses lead to the accumulation of plastic deformation on the underlying surface, followed by surface fatigue delamination. Furthermore, Figure 15 shows fluid-looking, smooth areas at the base of the wear track, indicating the formation of a mechanically mixed layer (MML) [34]. This layer consists of a mixture of oxide and metal that has redistributed on the surface as a result of plastic deformation and heating; although it provides a partially protective effect, it is known that this layer is not sufficiently stable in pure aluminum [35].

Wear surface images of 10 wt.% RMEA with different magnifications are given in Figure 15c,d. The figure reveals a distinct surface crack network, with much denser and deeper linear grooves at the base of the wear channel, in contrast to the pure Al sample. This morphology points to two critical interpretations.

For the first one, the hard WMoNb particles dispersed in the matrix after MA increased local hardness, restricting dislocation movement; this restriction increased surface brittleness under thermal and mechanical cycling, thus facilitating crack formation. The other, insufficient sintering bonding: the dark pits observed in some areas represent weak interparticle bonding regions that arise when the oxide layers formed during the MA process are not removed during sintering.

Although parallel linear grooves are preserved, the fluid MML structure, which is prominent in pure Al, is largely lost in this sample. This is because WMoNb particles inhibit MML formation by hindering the surface fluidity of the matrix. The dark, pitted morphological areas observed, particularly in the left central region, represent the emergence of residual porosities formed during the high-energy ball milling process, and these structures constitute preferred nucleation points for crack propagation paths.

The absence of large bright adhesion areas, unlike in the RMEA sample, indicates that RMEO reinforcement also provides a significant protective/hardening effect on the surface. In the 500× image (Figure 15f), a more complex surface character emerges. In addition to regular abrasive scratches, there are interspersed white, fine, and branched (dendritic-like) structures and dark, rough areas. This appearance points to a complex wear mechanism where both a limited level of adhesive component (white thin stripes, possibly a tribo-oxide layer or limited material transfer) and a dominant abrasive/delamination component coexist. Such fine, branched, white structures are generally associated in the literature with a protective tribo-oxide (tribofilm/glaze layer) layer formed during friction, and this layer is reported to play a role in enhancing wear resistance.

When the three 3D profilometer analyses in Figure 16 are evaluated together, it is clearly seen that the wear track depth and edge pile-up systematically decrease as we move from unreinforced pure Al to the 10 wt.% RMEA-reinforced composite and finally to the 10 wt.% RMEO-reinforced composite, thus gradually increasing the wear resistance. The total height difference is highest in the pure Al sample Figure 16a (R_t_ ≈ 392.1 μm; between +123.717 μm and −268.382 μm); in this sample, the deep wear valley, reaching −268 μm in particular, shows that the soft and ductile Al matrix undergoes severe adhesive wear and intense plastic deformation at the contact points with the mating surface, resulting in a large volumetric material loss. In the RMEA-reinforced composite Figure 16b, the total height difference decreased by approximately 42%, reaching R_t_ ≈ 228.8 μm (+111.512 μm/−117.309 μm); the hardness increase provided by the metallic refractory medium-entropy alloy particles limited both wear track depth and edge stacking, suppressing the plastic flow of the matrix. The lowest values were obtained for the 10 wt.% RMEO-reinforced composite Figure 16c (R_t_ ≈ 101.8 μm; +46.183 μm/−55.637 μm); in this sample, the wear track depth decreased by approximately four times compared to pure Al and by more than two times compared to RMEA. This significant improvement is in full agreement with previous SEM/EDS findings that oxidized RMEO reinforcement increases load-carrying capacity and limits material transfer not only through stiffness enhancement but also through a protective tribo-oxide layer formed during friction. Consequently, 3D profilometer data reveal a clear hierarchy in terms of wear resistance (Pure Al < 10% RMEA < 10% RMEO), quantitatively confirming that RMEO reinforcement provides the most effective tribological improvement in Al matrix composites. The four-fold reduction in wear track depth for the RMEO sample compared to pure Al, and more than two-fold reduction compared to RMEA, supports previous SEM/EDS findings indicating that oxidized RMEO reinforcement increases load-carrying capacity and limits material transfer not only through hardness enhancement but also through a protective tribo-oxide layer formed during friction.

The rapid transient increase in the COF within the first 0–100 m followed by stabilization represents a transition where wear is described as moving from an unpredictable level to a more predictable level. The pure aluminum sample’s rapid attainment of its peak COF (≈0.52) within approximately 50 m is a direct result of the low-hardness pure aluminum’s inability to resist rapid plastic deformation at the onset of contact. The sample containing 10% RMEA exhibited a significantly delayed run-in process (≈150 m) compared to all other samples. This unique behavior can be explained by the hard refractory particles (W, Mo, Nb), which are thought to prevent asperite flattening at the contact interface, and the high RMEA content, confirmed by SEM analysis, suppressing MML and thus delaying the surface’s tribological stability. It can be said that hard oxide phases (such as Mo_4_O_11_, NbO_0.76_) create an effective dispersion/load-carrying effect within the matrix, and in accordance with Archard’s law (wear ∝ 1/hardness), higher hardness correlates with lower wear loss [36]. At the same reinforcement ratio, RMEO consistently offers a lower COF than RMEA. This directly supports the interpretation that oxidized reinforcement reduces friction more effectively by forming a lubricating/protective tribo-oxide layer during friction. This observation is consistent with, though not independently confirmed by, the proposed protective tribo-oxide mechanism, since the lower COF alone does not verify that a continuous, adherent oxide layer—rather than a discontinuous or debris-mediated oxide contribution—was responsible for the friction reduction. A more definitive mechanical attribution would require complementary evidence, such as continuous morphological coverage in SEM or phase-specific identification of the tribo-oxide via micro-Raman/XRD analysis of the worn surface. Wear loss also shows a trend parallel to the COF: pure Al has the highest loss (131 mg), while the loss decreases as the reinforcement ratio increases. In the RMEO series, the lowest wear loss is achieved at 10% RMEO (52 mg); this corresponds to a reduction of approximately 60% compared to pure Al. Again, at the same ratios, RMEO provides lower wear loss than RMEA, which confirms the superior wear resistance of the oxidized reinforcement. While the COF shows a gradual, linear decrease (Δμ ≈ 0.038/wt.%) with increasing RMEA content from 2.5% to 7.5%, a non-linear, threshold-based, and dramatic decrease, however, is observed at the transition to 10% RMEA. This can be explained by the fact that pure Al (μ = 0.479) and 2.5% RMEA (μ = 0.449) correspond to a severely adhesive wear regime, while 10% RMEA (μ = 0.225) corresponds to a significantly milder wear regime. This transition is also quantitatively supported by a 56.3% decrease in the h_max_ value in the profilometer data. Comparative analysis of the cumulative wear loss (Figure 18) and the evolution of the friction coefficient (Figure 17) depending on the sliding distance reveals a strong and mutually consistent tribological response in all Al–WMoNb composite compositions. This can be fundamentally explained by the effect of hard oxide reinforcement on mechanical properties. The reason for the performance increase in RMEO may not simply be the presence of oxides, but specifically the Mo_4_O_11_/NbO_0.76_ mixed oxide shell + metallic W core–shell architecture.

## 5. Conclusions

Equiatomic WMoNb RMEA powder, produced by 150 h high-energy ball milling, formed a single-phase BCC solid solution (~22.1 nm crystallite size; ΔS_mix_ = 9.13 J/mol·K, ΔH_mix_ = −4.44 kJ/mol), consistent with MEA formation. Oxidation at 650 °C/4 h selectively converted Mo and Nb into Mo_4_O_11_ and NbO_0.76_ while W remained metallic, yielding a core–shell RMEO structure. SEM/EDS confirmed homogeneous distribution of both reinforcements in the Al matrix at all ratios (2.5–10 wt.%), with the RMEO series showing a proportionally higher surface oxide network at increasing content.

Dry sliding wear tests showed that both reinforcements reduced friction and wear loss relative to pure Al (COF = 0.479, wear loss = 131 mg, hardness = 61 HB) in a dose-dependent manner. At 10 wt.% RMEA, the COF and wear loss decreased to 0.225 and 68 mg (53% and ~48% reduction), with hardness rising to 114 HB. RMEO reinforcement outperformed RMEA at every ratio: at 10 wt.%, the COF and wear loss dropped to 0.192 and 52 mg (~60% reduction in both), with the highest hardness recorded (132 HB). This superior behavior is attributed to the combined hardening effect of the mixed oxide shell and the formation of a protective tribo-oxide layer that limited adhesive transfer and stabilized friction.

Three-dimensional profilometry corroborated these trends, showing a ~2-fold reduction in maximum wear track depth for RMEA and more than a ~4-fold reduction for RMEO relative to pure Al. Worn-surface SEM analysis confirmed a corresponding wear-mechanism transition: severe adhesive wear with a fluid mechanically mixed layer (MML) in pure Al, three-body abrasion with fatigue-induced delamination in RMEA composites, and a mixed abrasive/tribo-oxide-mediated regime in RMEO composites.

Overall, both WMoNb RMEA and its oxidized RMEO derivative are effective reinforcement phases for improving the wear resistance of Al matrix composites, with RMEO offering superior friction and wear performance at every reinforcement level. These results support controlled, selective oxidation of refractory medium-entropy alloy powders as a viable strategy for tailoring the tribological response of lightweight Al matrix composites. Future work should address the long-term thermal stability of the tribo-oxide layer under varying load, velocity, and temperature conditions, as well as the mechanical properties (tensile strength, fracture toughness) of the developed composites. Future studies should prioritize wear tests with multiple replicates and a broad range of parameters, verification of the tribo-oxide layer via Raman/XPS, comprehensive mechanical characterization, and investigation of the thermal stability of the RMEO reinforcement.

## Figures and Tables

**Table 1:** Crystallite size of peaks.

B (FWHM) (rad)	θ (Degree)	D (nm)
0.062	18.085	13,413
0.023	20.235	36,248
0.056	21.5	14,963
0.028	29.205	30,115
0.075	37.105	11,111
0.031	43.455	26,751
Average D:		22.1

**Table 2:** Key differences between the two XRD patterns.

Feature	Unoxidized (150 h MA)	Oxidized (650 °C/4 h)
Main phase	Single-phase BCC solid solution (W + Mo + Nb)	W (metallic, BCC) + Mo_4_O_11_ + NbO_0.76_
Number/character of peaks	4 BCC peaks (110, 200, 211, 220), broad and overlapping	Segregated oxide peaks + residual BCC W peak
Elemental state	W, Mo, Nb homogenized in the same lattice	Mo and Nb selectively oxidized, W remains metallic
Structural implication	Nanocrystalline RMEA powder successfully synthesized	Onset of core–shell architecture via controlled selective oxidation

**Table 3:** Data from 3D profilometer analysis of the samples.

Samples	Maximum Groove Depth, h_max_ (µm)	Pile-Up Height, h_pile-up_ (µm)	Peak-to-Valley Amplitude, R_t_ (µm)	Reduction Factor vs. Pure Al
Pure Al	−268	+124	392	-
RMEA (10 wt.%)	−117.3	+111.5	229	2.28x
RMEO (10 wt.%)	−55.6	+46.2	101.8	4.82x

**Table 4:** Average friction coefficient values of the samples, wear loss, hardness, and percentage decreases compared to the pure Al sample.

Samples	COF (Average)	Wear Loss (mg) (1000 m)	Hardness (HB)	Decrease (%)
Pure Al	0.47876	−131	61 (±%5)	(None)
2.5 wt.% RMEA	0.44855	−121	63 (±%5)	−6.3
5 wt.% RMEA	0.40149	−106	65 (±%5)	−16.1
7.5 wt.% RMEA	0.36194	−98	77 (±%5)	−24.4
10 wt.% RMEA	0.22485	−68	114 (±%5)	−53
2.5 wt.% RMEO	0.32374	−92	80 (±%5)	−32.38
5 wt.% RMEO	0.30431	−87	82 (±%5)	−36.44
7.5 wt.% RMEO	0.25903	−72	95 (±%5)	−45.9
10 wt.% RMEO	0.19176	−52	132 (±%5)	−59.95

## Data Availability

The original contributions presented in this study are included in the article. Further inquiries can be directed to the corresponding author.

## Abbreviations

The following abbreviations are used in this manuscript:

HEA	High-Entropy Alloy
RMEA	Refractory Medium-Entropy Alloy
RMEO	Refractory Medium-Entropy Oxide
MEA	Medium-Entropy Alloys
PBF-LB/M	Laser Powder Bed Fusion
FSP	Friction Stir Processing
BRP	Ball-To-Powder Weight Ratio
EDS	Energy-Dispersive X-Ray Spectroscopy
FWHM	Full Width at Half Maximum
BCC	Body-Centered Cubic
HEBM	High-Energy Ball Milling
MA	Mechanical Activation
MML	Mechanically Mixed Layer
